# Dissecting G-protein signaling pathways in the fruit pathogen *Penicillium expansum*: implications for pathogenesis and patulin production

**DOI:** 10.1186/s43897-025-00211-w

**Published:** 2026-04-08

**Authors:** Yong Chen, Dongying Xu, Mengyang Xing, Tong Chen, Boqiang Li, Shiping Tian

**Affiliations:** 1 State Key Laboratory of Plant Diversity and Specialty Crops, Institute of Botany, Chinese Academy of Sciences, Beijing, 100093 China; 2 China National Botanical Garden, Beijing, 100093 China; 3 University of Chinese Academy of Sciences, Beijing, 100049 China

**Keywords:** *Penicillium expansum*, Pathogenicity, Patulin production, G-protein signaling pathways, Fungal hyphal polarity

## Abstract

*Penicillium expansum* causes blue mold in various fruits and produces patulin, a mycotoxin of significant economic and food safety concern. Fungal infection relies on sensing and adapting to environmental cues via G-protein signaling pathways. Despite its importance, the understanding of how these pathways contribute to the infection process and patulin biosynthesis of *P. expansum* remains limited. Here, we characterized five G protein subunits (PeGαⅠ, PeGαⅡ, PeGαⅢ, PeGβ, and PeGγ), all highly expressed during hyphal growth and fruit infection. Deletion of these G protein genes leads to various defects in hyphal growth, virulence, and patulin production. Notably, Δ*PeGαⅠ*, Δ*PeGβ*, and Δ*PeGγ* mutants showed severe defects, with minimal disease symptoms in early infection stages and at least a 50% decrease in patulin production. These mutants displayed straight hyphal growth, reduced branching, and significantly decreased vitality. RNA sequencing analysis revealed that this abnormal growth is governed by pathways associated with fungal hyphal polarity. Furthermore, PeGαⅠ and PeGαⅢ are crucial for cAMP-PKA signaling, while PeGβ and PeGγ modulate MAPK signaling, both pathways influencing virulence and patulin production. Together, these findings provide evidence that G proteins regulate virulence and patulin production through the regulation of downstream pathways and their broader effects on fungal hyphal polarity.

**Supplementary Information:**

The online version contains supplementary material available at 10.1186/s43897-025-00211-w.

## Core

This study systematically characterized five G protein subunits in *P. expansum*, revealing their pivotal roles in regulating hyphal growth, virulence, and patulin production through distinct signaling pathways. Specifically, PeGαⅠ and PeGαⅢ primarily function via the cAMP-PKA pathway, whereas PeGβ and PeGγ modulate the MAPK signaling pathway, both of which significantly influence virulence and patulin biosynthesis. Furthermore, PeGαⅠ, PeGβ, and PeGγ exhibit broader regulatory effects on fungal hyphal polarity, leading to decreased vitality, which could impact growth and pathogenicity.

## Gene & accession numbers

Information for the genes in this article can be found in the NCBI database, accession numbers: PeGαI (XP_016596079.1), PeGαII (XP_016601136.1), PeGαIII (XP_016596161.1), PeGβ (XP_016601560.1), PeGγ (XP_016595165.1), PePkaA (XP_016599345.1), PePkaB (XP_016600505.1), PeSlt2 (XP_016593251.1), PeFus3 (XP_016600869.1), PeHog1 (XP_016594988.1).

## Introduction

*Penicillium expansum*, a prevalent saprophytic fungus, is a significant postharvest pathogen causing blue mold, leading to substantial economic losses in the fruit industry (Li et al. 2020a). This pathogen infects fruit tissues through wounds during harvesting, handling, and storage, resulting in tissue decay (Tahtah et al. 2023). Moreover, *P. expansum* produces patulin, a mycotoxin harmful to humans and animals, with severe health consequences such as nausea, gastrointestinal damage, kidney impairment, cancer, and developmental defects (Li et al. 2019; Jimdjio et al. 2021; Llobregat et al. 2024). Therefore, controlling the growth and patulin production of *P. expansum* is crucial for ensuring food safety and protecting public health. As a necrotrophic pathogen, *P. expansum* infects fruit wounds by secreting virulence factors that kill host cells, enabling the fungus to access nutrients from dead tissues (Li et al. 2020a). Recent research has revealed that the production of these virulence factors is regulated by internal regulatory elements and external environmental cues, including transcription factors such as PePacC in the pH signaling pathway (Chen et al. 2018), PeMetR in sulfur metabolism (Chen et al. 2021), and PeAP1 in the antioxidant pathway (Chen et al. 2023). However, the mechanisms by which *P. expansum* senses host nutrients and environmental cues, and how these signals are transduced intracellularly to regulate transcription factors, remain poorly understood and require further investigation.

The heterotrimeric guanine nucleotide-binding protein (G protein) signaling pathway plays a crucial role in signal transduction in eukaryotic organisms. Comprising Gα, Gβ, and Gγ subunits, G proteins are activated by G-protein-coupled receptors (GPCRs) to initiate cellular responses (Xu et al. 2018; Xie et al. 2022). Upon ligand binding to GPCRs, they act as guanine nucleotide exchange factors, facilitating the exchange of Gα-bound guanosine diphosphate (GDP) for guanosine triphosphate (GTP) and leading to the dissociation of the Gα subunit from the Gβγ complex (Park et al. 2020). The resulting Gα-GTP and Gβγ heterocomplexes then transmit signals to downstream effectors through various pathways, including the adenylate cyclase-cAMP-dependent protein kinase A (PKA) pathway, the inositol triphosphate (IP3)-Ca^2^⁺-diacylglycerol-dependent protein kinase C (PKC) pathway, and the mitogen-activated protein kinase (MAPK) pathway (Desale et al. 2021). These pathways enable fungi to regulate cellular functions, metabolism, and development in response to environmental stimuli, thereby enhancing their survival, propagation, and virulence (Brown et al. 2018; Xie et al. 2022).

Filamentous fungi typically possess three conserved Gα subunits, one Gβ protein, and one Gγ protein, with the Gα subunits classified into three distinct groups (Li et al. 2007). Class I and III Gα proteins feature a conserved N-terminal myristoylation site (MGXXXS), while only class I Gα proteins have an additional C-terminal pertussis toxin-labeling site (CXXX). In contrast, class II Gα proteins lack these characteristic sites and exhibit more diverse functional roles (Tong et al. 2022). The Gβ subunit, a well-characterized WD40 repeat-containing protein, is among the most evolutionarily conserved and complex receptor effector signaling mechanisms in eukaryotes (Miller et al. 2016). It forms obligate heterodimers (Gβγ) with the smaller Gγ subunit. Heterotrimeric G proteins serve as central regulators in fungal pathogens, governing essential biological processes including signal transduction, developmental regulation, virulence, and mycotoxin biosynthesis. They are central in coordinating major life cycle transitions. For instance, in *Phytophthora infestans*, Gα, Gβ, and Gγ regulate zoospore motility and sporangium formation (Van den Hoogen et al. 2018). *Chaetomium globosum* relies on the Gα-cAMP signaling pathway for perithecium formation (Hu et al. 2018a), while *Aspergillus nidulans* utilizes the Gα subunit GanB to modulate asexual conidiation and germination through the cAMP/PKA pathway in response to glucose (Chang et al. 2004). In terms of pathogenicity, different regulatory mechanisms are observed among these subunits. In *Valsa mali*, Gα proteins Gvm2 and Gvm3 enhance virulence by regulating the production of cell wall-degrading enzymes (Song et al. 2017). *Botrytis cinerea* relies on the Gβ subunit Bcgb1 to coordinate cAMP and MAPK signaling pathways for full virulence (Tang et al. 2021). Meanwhile, the Gγ subunit Stgg1 is essential for the pathogenicity of *Setosphaeria turcica*, making it a promising target for disease management (Li et al. 2018). Mycotoxin production is also influenced by G-protein signaling networks. In *Aspergillus flavus*, the Gα subunit GpaB suppresses aflatoxin biosynthesis by modulating the cAMP signaling pathway (Liu et al. 2018). Similarly, in *Aspergillus fumigatus*, the Δ*gpaB* mutant reduces gliotoxin production through PKA/PKC-dependent pathways (Choi et al. 2020). In *Gibberella zeae*, both the Gα subunit GzGPA1 and the Gβ subunit GzGPB1 act as negative regulators of trichothecene and zearalenone biosynthesis (Yu et al. 2008). Despite these advancements, the precise mechanisms underlying G-protein-mediated regulation of fungal virulence and mycotoxin biosynthesis are yet to be fully understood.

In the present study, we characterized five G protein subunits in *P. expansum* and revealed their critical roles in regulating hyphal growth, virulence, and patulin production through distinct signaling pathways. PeGαⅠ, PeGβ, and PeGγ were also found to exert broad influences on fungal hyphal polarity, leading to diminished vitality that could detrimentally affect growth and pathogenicity.

## Results

### Identification of G protein subunits in *P. expansum*

The G protein subunits in *P. expansum* were identified through BLASTp homology searches using *Neurospora crassa* G protein subunit sequences from the NCBI database. *P. expansum* possesses five homologs: PeGαI, PeGαII, PeGαIII, PeGβ, and PeGγ, with similarities of 93.48%, 52.51%, 76.19%, 82.72%, and 64.52% to their respective components. Protein domain analysis revealed that all three Gα subunits contain conserved G_alpha domains, while PeGβ and PeGγ possess conserved WD40 repeats and G_gamma domains, respectively (Fig. S1). Phylogenetic analysis indicated that the five G protein subunits were located in distinct branches, with each subunit and its homologs from various species showing close relationships (Fig. S1). Notably, *P. expansum* exhibited the closest evolutionary relationship to *A. nidulans*. These findings suggest a significant conservation of G protein subunits during evolution.

### Expression patterns of G protein genes at different developmental stages

**Fig. 1:**
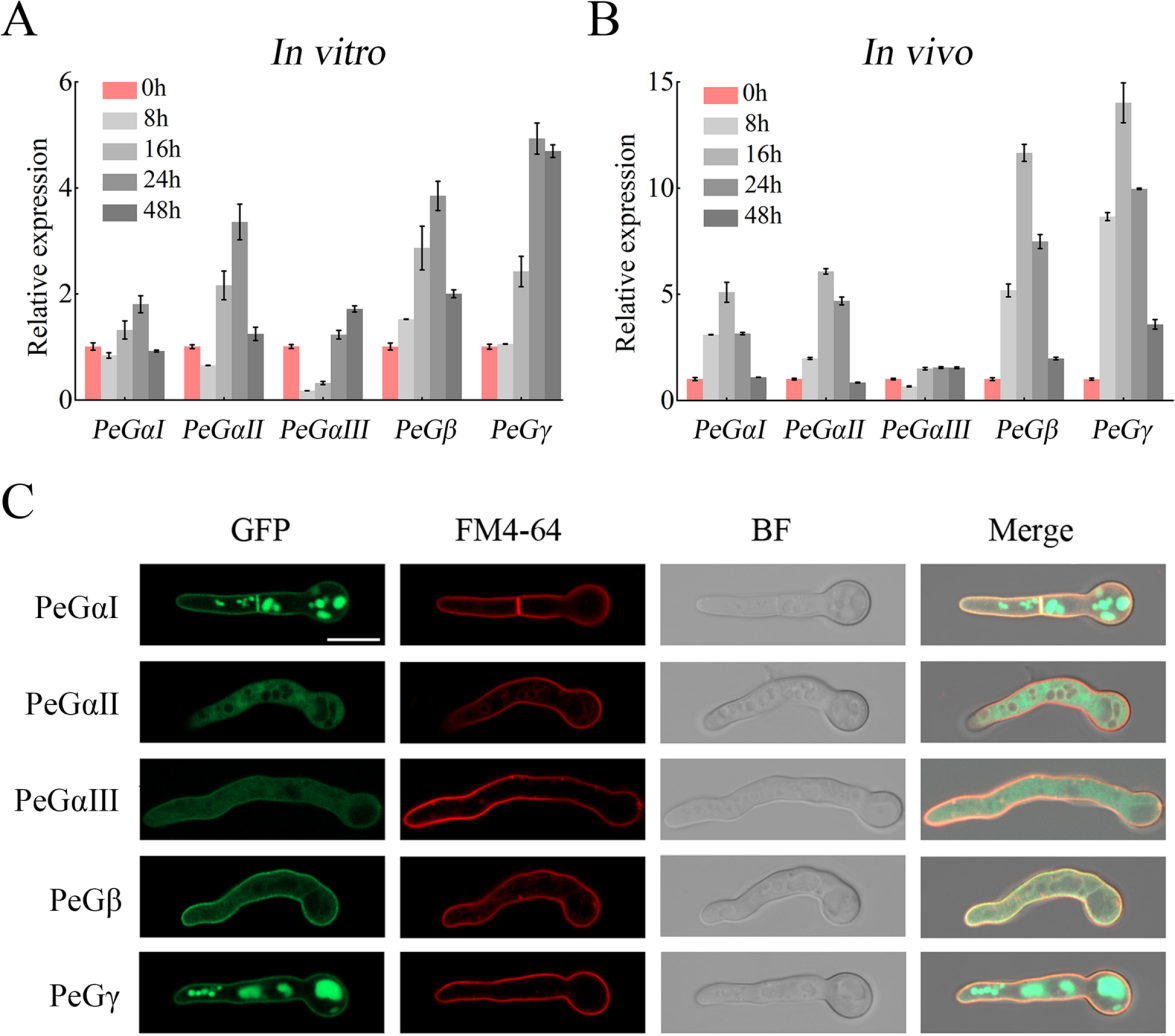
Gene expression and subcellular localization of G protein subunits in *P. expansum*. **A**, **B**. Phase-specific expression of *PeGαⅠ*, *PeGαⅡ*, *PeGαⅢ*, *PeGβ*, and *PeGγ*. Gene expression was quantified by RT-qPCR using cDNA from samples during vegetative growth (**A**, from ungerminated conidia to fungal cells at 48 hpi) and infectious growth (**B**, from ungerminated conidia to in planta fungal cells at 48 hpi). Relative expression levels were normalized to *β-tubulin* as an internal control. Data represent mean ± SEM (*n* = 3). **C**. Subcellular localization of PeGαⅠ, PeGαⅡ, PeGαⅢ, PeGβ, and PeGγ. The PeGαⅠ::eGFP, PeGαⅡ::eGFP, PeGαⅢ::eGFP, PeGβ::eGFP, and PeGγ::eGFP strains were cultured in CY media for 13–15 h and visualized for GFP fluorescence. Cell membranes were stained with FM4-64. Scale bar = 20 μm

Gene expression patterns of G protein subunits in *P. expansum* were analyzed using RT-qPCR during vegetative growth and host infection stages. *PeGαI*, *PeGαII*, *PeGβ*, and *PeGγ* showed an initial increase followed by a decrease during vegetative growth, peaking at 24 h post-inoculation (hpi) (Fig. 1A). In contrast, *PeGαIII* exhibited low transcript levels during spore germination and early hyphal growth, gradually increasing later. During the infection stage, *PeGαI*, *PeGαII*, *PeGβ*, and *PeGγ* showed a similar trend, peaking at 16 hpi, while *PeGαIII* showed a slight increase in expression (Fig. 1B). These results indicate potential diverse functions of G protein subunits across distinct developmental stages.

### Subcellular localizations of G protein subunits

To investigate the subcellular localization of G protein subunits, eGFP-tagged fusion constructs were created for each subunit and introduced into the wild-type (WT) strain. Two or more independent GFP fusion transformants were obtained for each subunit (Fig. S2). The transformants (PeGαI::eGFP, PeGαII::eGFP, PeGαIII::eGFP, PeGβ::eGFP, and PeGγ::eGFP) were cultured in CY media with shaking for 13–15 h, stained with FM4-64 to label the cell membrane, and imaged using a laser confocal microscope. The results revealed significant cell membrane localization for PeGαI, PeGβ, and PeGγ, a membrane-cytosol co-localization pattern for PeGαIII, and predominantly cytosolic localization for PeGαII (Fig. 1C). These findings demonstrate distinct subcellular localization patterns of G protein subunits that may be functionally relevant.

### G protein subunits are important for vegetative growth and conidiation

In order to investigate the functional roles of G protein subunits in *P. expansum*, deletion mutants Δ*PeGαI*, Δ*PeGαII*, Δ*PeGαIII*, Δ*PeGβ*, and Δ*PeGγ* were generated using the ATMT method (Fig. S3A). All mutants were verified by PCR and further confirmed through Southern blot analysis (Fig. S3B, C). Additionally, complementary strains for each gene, designated as *PeGαI*^*C*^, *PeGαII*^*C*^, *PeGαIII*^*C*^, *PeGβ*^*C*^, and *PeGγ*^*C*^, were successfully constructed (Fig. S3D).

**Fig. 2:**
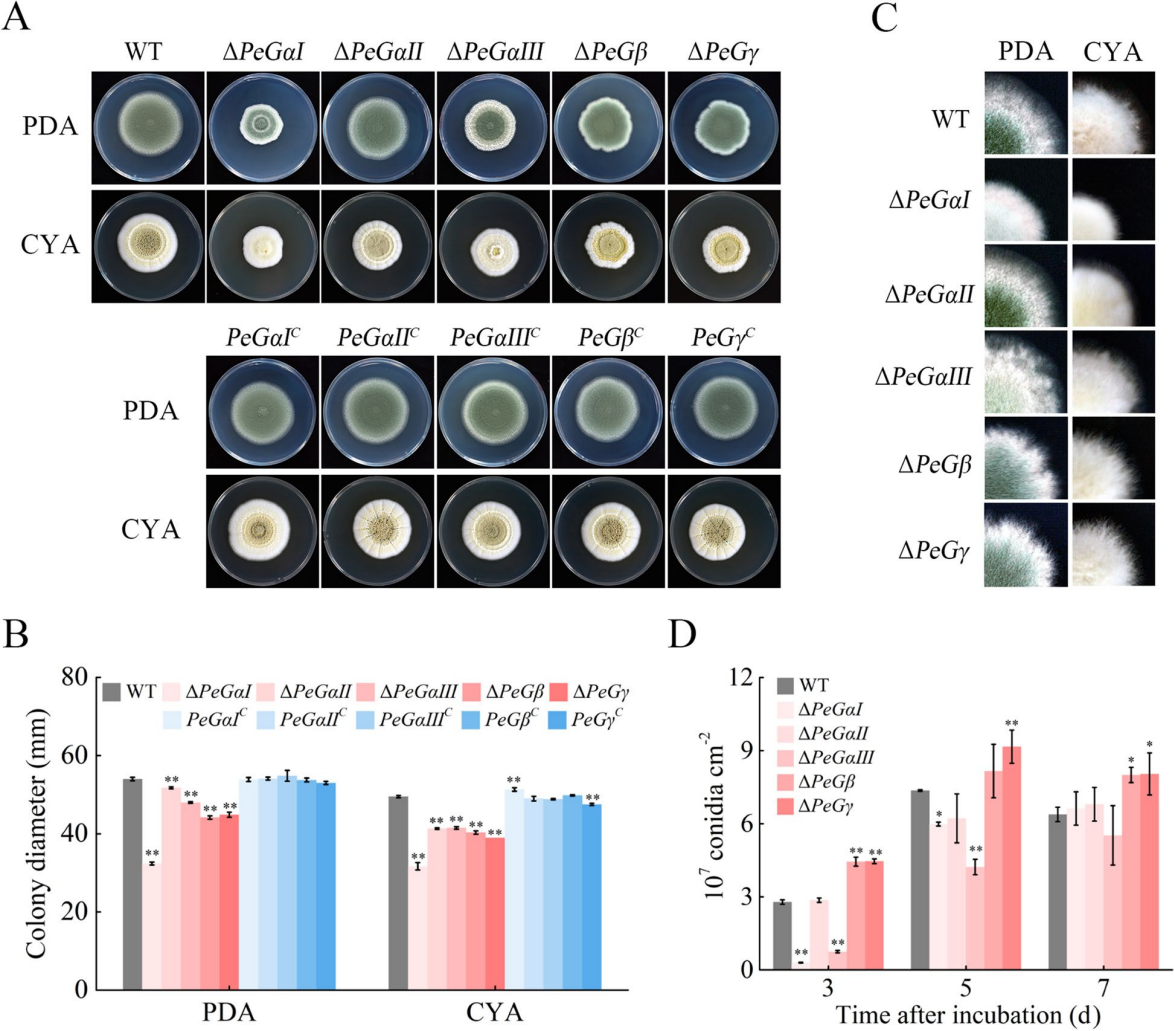
G protein subunits regulate vegetative growth and conidiation of *P. expansum*. **A** Colony morphologies of the WT, deletion mutants, and complementary strains of G protein genes on PDA and CYA media at 7 dpi. **B** Colony diameters of the tested strains at 7 dpi. Error bars represent standard deviation (SD) of the mean (*n* = 4). **C** Colony edge morphologies of the tested strains on PDA at 3 dpi. **D** Conidiation of the tested strains on PDA at 7 dpi. Data represent the mean ± SEM (*n* = 3). *P < 0.05, **P < 0.01

To assess the involvement of G protein subunits in vegetative growth and development, hyphal extension and conidiation of the mutants were examined over a 7-day period. Deletions of G protein subunit genes resulted in varying degrees of growth reduction, with ∆*PeGαI*, ∆*PeGβ*, and ∆*PeGγ* strains exhibiting decreases in growth by 40.05%, 18.21%, and 16.90% respectively at 5 days post-inoculation (dpi) (Fig. 2A, B). Notably, irregular wavy colony edges were observed in ∆*PeGβ* and ∆*PeGγ* strains (Fig. 2C). Conidiation was also significantly impacted, being reduced in the ∆*PeGαI* and ∆*PeGαIII* strains, while increased in the ∆*PeGβ* and ∆*PeGγ* strains (Fig. 2D). Complementary strains restored colony morphology and hyphal growth to the WT level. These results highlight the crucial roles of PeGαI, PeGαIII, PeGβ, and PeGγ in regulating hyphal growth and conidiation in *P. expansum*.

### G protein subunits contribute to fungal stress responses and nutrient sensing

To examine the roles of G protein subunits in stress responses in *P. expansum*, mutant strains were tested under various stress conditions (Fig. S4A). Δ*PeGαI* and ∆*PeGαIII* exhibited reduced growth inhibition under light exposure and NaCl treatments, indicating decreased sensitivity to light and osmotic stress. In contrast, these mutants showed enhanced growth inhibition under Congo Red treatment, suggesting increased sensitivity to cell wall stress. ∆*PeGβ* and ∆*PeGγ* showed similar response patterns, displaying decreased sensitivity to light, low pH, and osmotic stress, but increased sensitivity to high pH and cell wall stress. In nutrient sensing assays, Δ*PeGαI* showed reduced growth inhibition under different nitrogen sources (peptone and NH_4_Cl), while ∆*PeGαIII* exhibited decreased growth inhibition across multiple nutritional conditions (Fig. S4B). ∆*PeGβ* and ∆*PeGγ* showed moderate responses to nutritional conditions. Overall, PeGαI, PeGαIII, PeGβ, and PeGγ all contribute to stress responses in *P. expansum*, with PeGαI and PeGαIII playing particularly significant roles in nutrient sensing.

### G protein subunits are crucial for the virulence of *P. expansum*

**Fig. 3:**
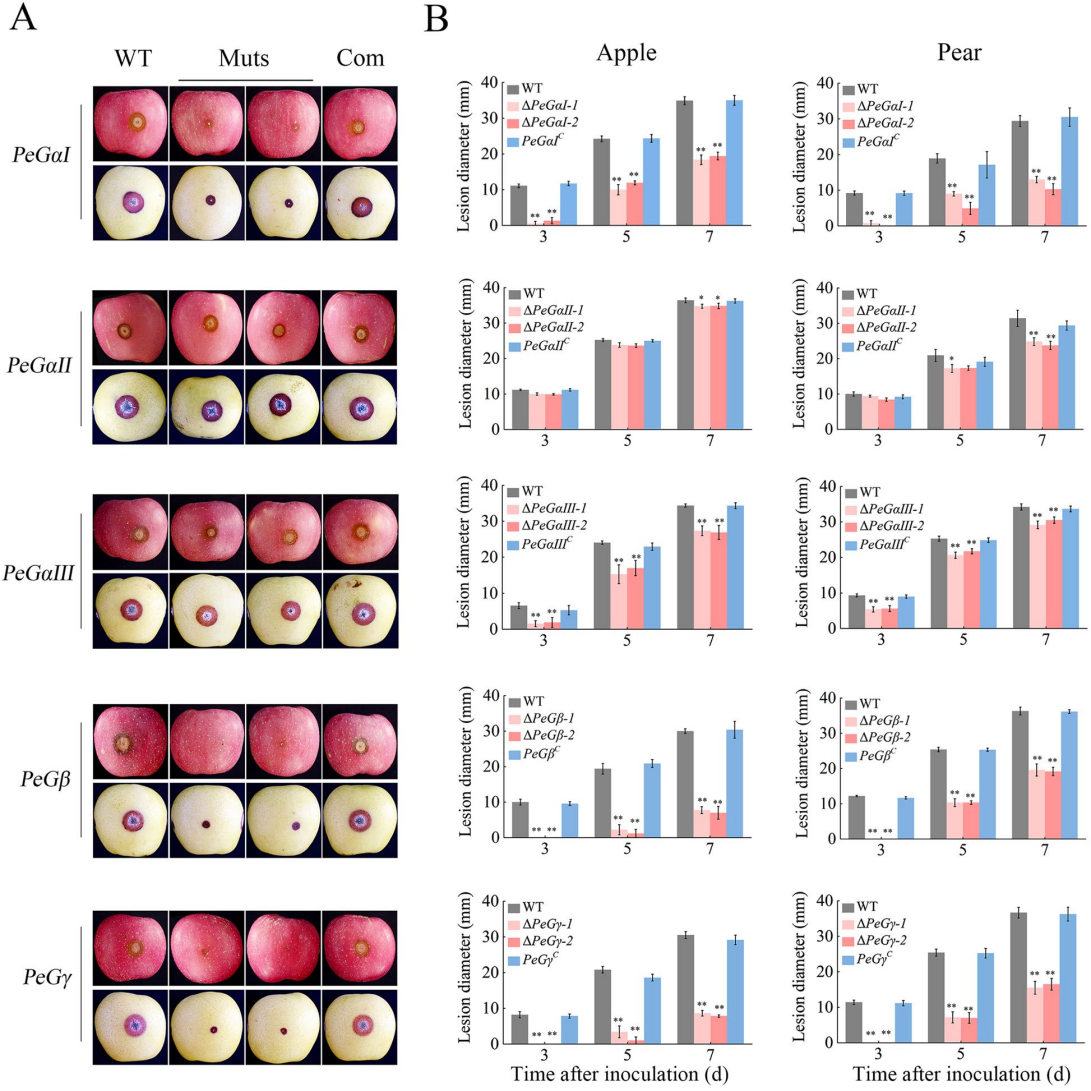
G protein subunits plays an essential role in the pathogenicity of *P. expansum*. Disease symptoms (**A**) and lesion diameters (**B**) on apple and pear fruit inoculated with conidia of the WT, deletion mutants, and complementary strains of G protein genes. Photographs were taken at 5 dpi. Lesion diameters were measured at 3, 5, and 7 dpi. Muts, mutant strains; Com, complementary strains. Error bars represent SD of the mean (*n* = 3). *P < 0.05, **P < 0.01

To evaluate the influence of G protein subunits on the pathogenicity of *P. expansum*, conidial suspensions of WT, G protein mutants, and complementary strains were inoculated onto apple and pear fruits, and disease progression was monitored. ∆*PeGαI*, ∆*PeGβ*, and ∆*PeGγ* displayed significantly reduced pathogenicity, exhibiting no rot symptoms at 3 dpi and disease severity reductions of 51–74%, 59–94%, and 72–95%, respectively, at 5 dpi compared to the WT strain (Fig. 3). ∆*PeGαIII* also demonstrated decreased pathogenicity, particularly during early infection, with a 40–76% reduction in lesion diameters at 3 dpi. Conversely, Δ*PeGαII* displayed minor pathogenicity defects, with a slight reduction observed during later infection stages. Reintroduction of the G protein genes complemented the reduced virulence (Fig. 3). These results indicate that all five subunits are necessary for *P. expansum* pathogenicity in fruit, with PeGαI, PeGβ, and PeGγ as key regulators.

### PeGα, PeGβ, and PeGγ regulate fungal hyphal polarity and vitality

**Fig. 4:**
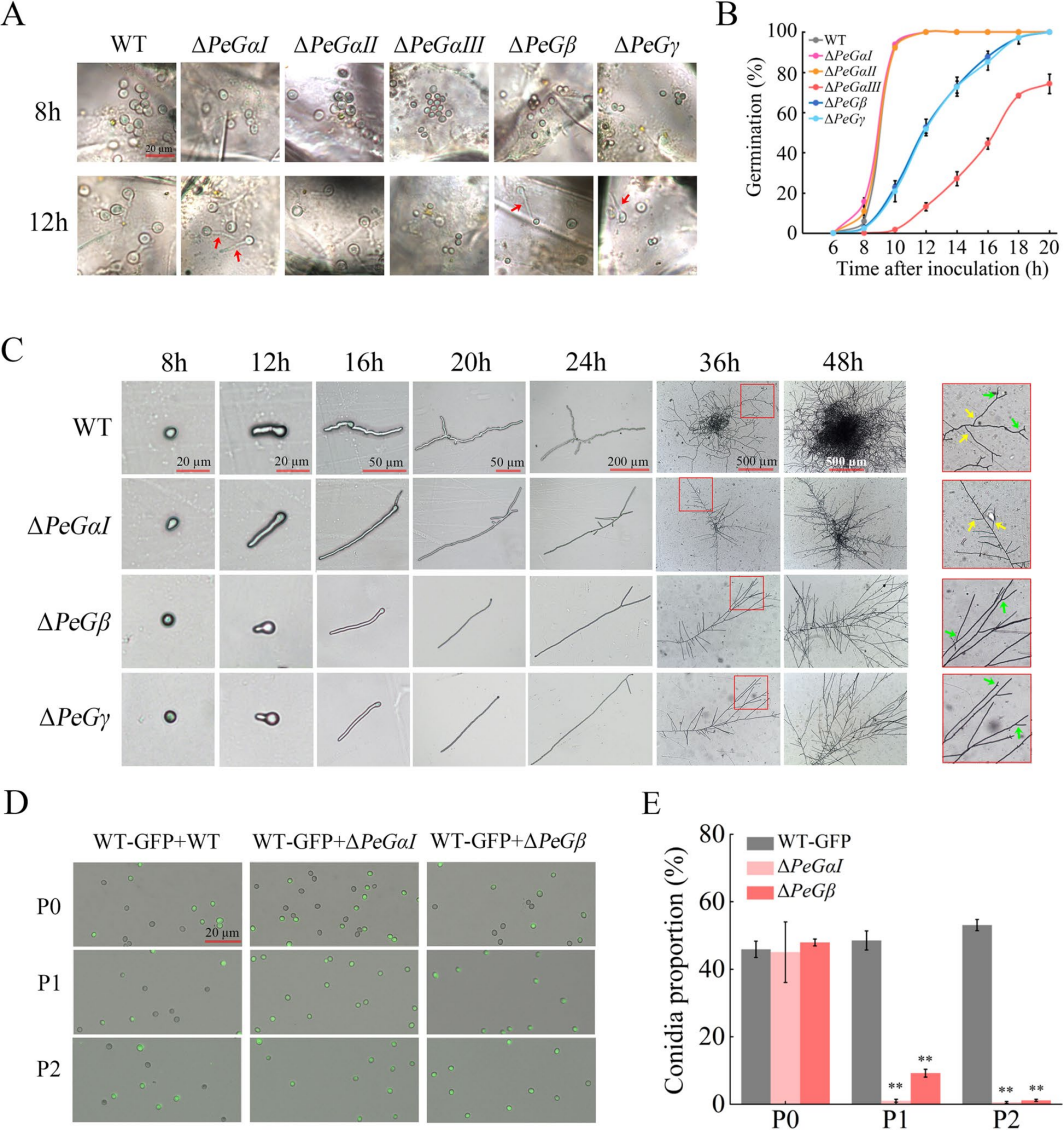
PeGαⅠ, PeGβ, and PeGγ regulate hyphal polarity and vitality of *P. expansum*. **A** Conidial germination of WT and deletion mutants of G protein genes on apple fruit wound sites. Red arrows indicate hyphae growing in a straight line. Scale bar = 20 μm. **B** Germination rates of the tested strains at 6–20 hpi. **C**. Conidial germination of the WT, Δ*PeGαⅠ*, Δ*PeGβ* and Δ*PeGγ* strains on PDA at 8–48 hpi. Green and yellow arrows indicate apical and lateral branches, respectively. **D**, **E** Co-incubation assays of the WT-GFP strain with the WT, Δ*PeGαⅠ*, Δ*PeGβ*, and Δ*PeGγ* strains. Conidia were mixed at a 1:1 ratio and co-cultured on PDA for 7 d. Conidia were harvested, examined microscopically (**D**), and quantified (**E**). P0, P1, and P2 denote the initial, first, and second passages, respectively. Error bars represent SD of the mean (*n* = 3). **P < 0.01

To investigate the regulatory mechanism of G protein subunits on the pathogenicity of *P. expansum*, spore germination of the mutants and WT strains on wounded apple fruits was assessed. WT spores exhibited normal germination at fruit wounds, achieving nearly 100% germination at 10 hpi (Fig. 4A, B). Δ*PeGαI* and Δ*PeGαII* showed similar germination rates to WT. In contrast, Δ*PeGαIII*, Δ*PeGβ*, and Δ*PeGγ* displayed significantly delayed spore germination, particularly Δ*PeGαIII*, which reached only 74.34% germination at 20 hpi. Notably, Δ*PeGαI*, Δ*PeGβ*, and Δ*PeGγ* spores demonstrated straight growth patterns at fruit wounds, differing from the wave-like germ tubes in the WT strain (Fig. 4A). Given that these mutants did not induce disease symptoms during the early stages of inoculation, it is hypothesized that their reduced pathogenicity may be associated with the straight growth of hyphae.

To further elucidate the abnormal growth patterns of these fungal strains, WT, ∆*PeGαI*, ∆*PeGβ*, and ∆*PeGγ* strains were inoculated on PDA plates, and spore germination and growth were continuously monitored. The WT strain exhibited normal germination, producing both apical branches (Fig. 4C, indicated by green arrows) and lateral branches (Fig. 4C, indicated by yellow arrows). At 36 hpi, the hyphae extended uniformly from the spore germination site, forming a reticulate structure. Remarkably, the ∆*PeGαI* strain displayed sustained straight growth with only lateral branches and an irregular rhomboid morphology (Fig. 4C). Conversely, both ∆*PeGβ* and ∆*PeGγ* strains predominantly showed apical branching with limited lateral branches, presenting an irregular broom-like morphology (Fig. 4C).

Co-cultivation experiments involving ∆*PeGαI*, ∆*PeGβ*, and WT-GFP strains in equal proportions revealed spore ratios of 1.03% and 9.20% for ∆*PeGαI* and ∆*PeGβ* strains, respectively, in the first generation (P1) (Fig. 4D, E). By the second generation (P2), spore production of both mutant strains decreased below 1%, while the WT control maintained a spore ratio of approximately 50%, indicating a notable decline in vitality of ∆*PeGαI* and ∆*PeGβ* strains. In conclusion, the abnormal growth patterns in ∆*PeGαI*, ∆*PeGβ*, and ∆*PeGγ* mutants may limit their ability to efficiently absorb nutrients from the surrounding hyphae, thus reducing their vitality and ultimately weakening their pathogenicity.

### G protein subunits are crucial for the patulin biosynthesis in vitro and in vivo

**Fig. 5:**
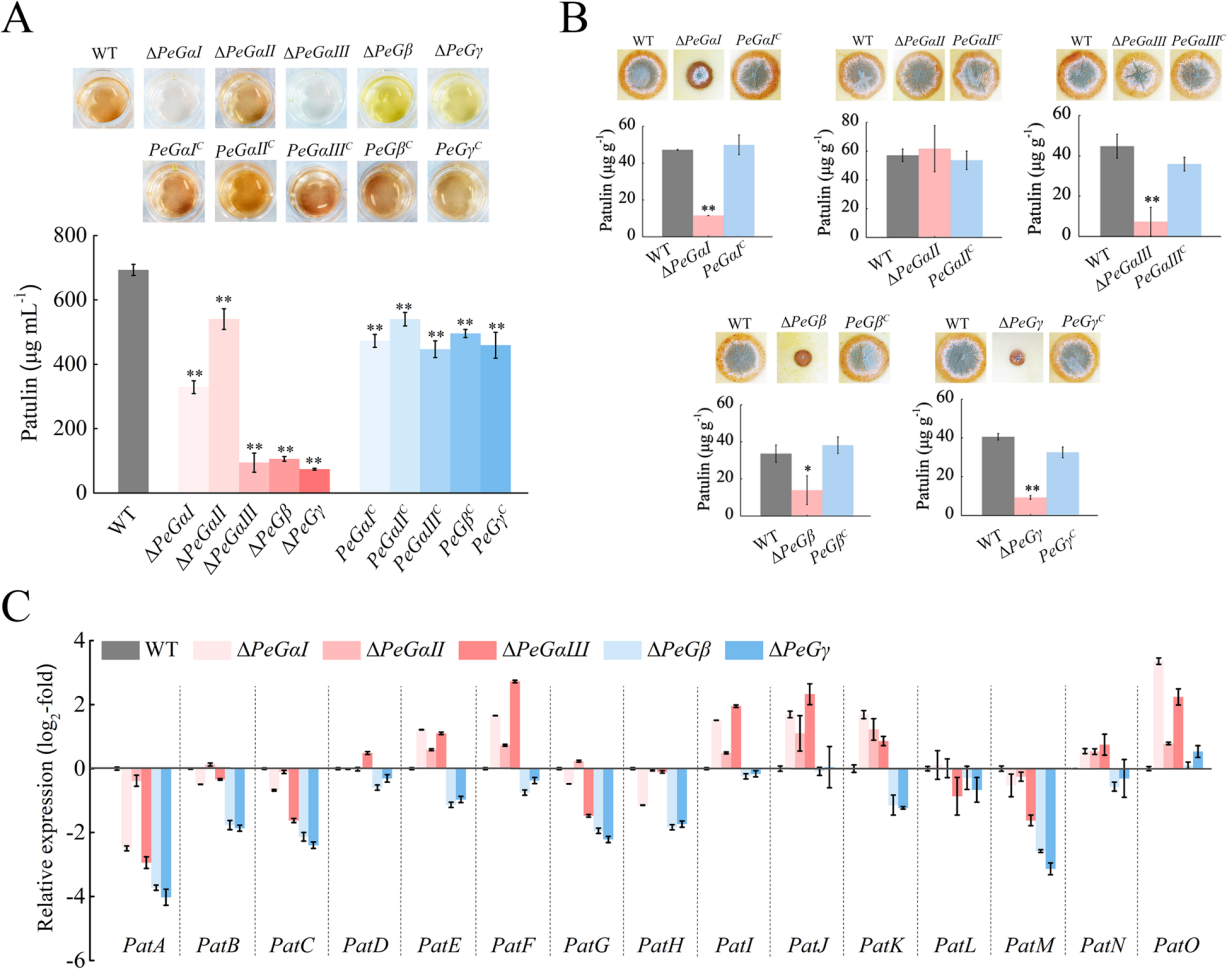
G protein subunits regulate the patulin biosynthesis of *P. expansum*. **A** Patulin production of the WT, deletion mutants, and complementary strains of G protein genes in vitro. Strains were cultured on CY media for 2 d, and filtrates were analyzed by HPLC. **B** Patulin production of the tested strains in vivo. Conidia were inoculated into pear fruit wounds and incubated at 25 °C for 7 d. Decayed tissue was collected for patulin analysis. **C** Expression analysis of patulin cluster genes in WT and deletion mutants of G protein genes. Mycelia from in vitro cultures were used for RNA extraction and RT-qPCR. Primers were from Li et al. (2015a). *β-tubulin* served as the internal control. Data represent mean ± SEM (*n* = 3). *P < 0.05, **P < 0.01

In order to examine the involvement of G protein subunits in patulin biosynthesis, the patulin production capabilities of WT, G protein mutants, and complementary strains were evaluated under both in vivo and in vitro conditions. The results showed a significant decrease in yellowish-brown pigment production in ∆*PeGαI*, ∆*PeGαIII*, ∆*PeGβ*, and ∆*PeGγ* mutants (Fig. 5A). These mutants also exhibited varying degrees of reduction in patulin production compared to the WT strain in CY media. Specifically, patulin production decreased by over 85% in ∆*PeGαIII*, ∆*PeGβ*, and ∆*PeGγ* mutants, while in ∆*PeGαI*, the reduction was approximately 50%. Additionally, patulin production was notably lower in G protein mutants (excluding Δ*PeGαII*) in the inoculated pear tissues (Fig. 5B). Complementation with G protein genes restored toxin production in both in vitro and in vivo settings (Fig. 5A, B). These findings highlight the critical regulatory roles of PeGαI, PeGαIII, PeGβ, and PeGγ in patulin biosynthesis.

Further analysis of the expression levels of all 15 genes within the patulin cluster revealed significant downregulation in all genes, except *PatO*, in ∆*PeGβ* and ∆*PeGγ* mutant strains (Fig. 5C). Conversely, in ∆*PeGαI* and ∆*PeGαIII* mutants, only 6 genes (*PatA*, *PatB*, *PatC*, *PatG*, *PatH*, and *PatM*) displayed significantly reduced expression levels. These results suggest distinct regulatory mechanisms for patulin biosynthesis among different G protein subunits, as indicated by the observed color changes in mutant strain pigments in CY media.

### PeGαI, PeGβ, and PeGγ modulate a series of genes associated with hyphal polarity growth pathways

**Fig. 6:**
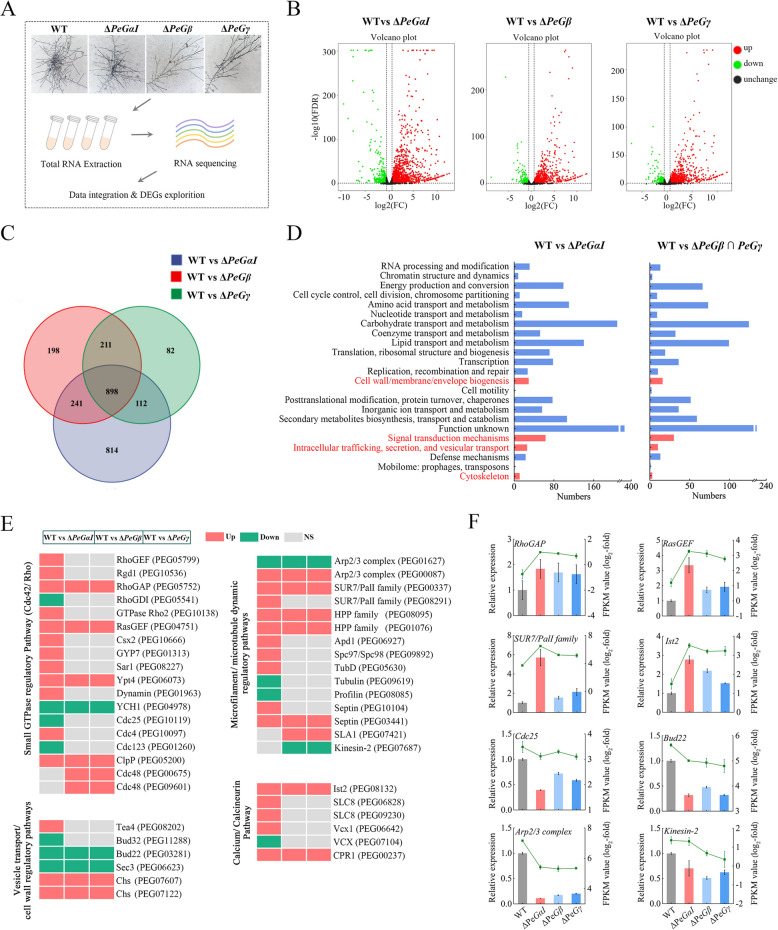
Differential gene expression analysis of WT, Δ*PeGαⅠ*, Δ*PeGβ* and Δ*PeGγ* strains. **A** Mycelia of WT, Δ*PeGαⅠ*, Δ*PeGβ* and Δ*PeGγ* strains cultured on PDA for 36 h were used for RNA-seq. DEGs were identified using FDR < 0.01 and fold change ≥ 1.5. **B** Volcano plot of gene expression patterns in Δ*PeGαⅠ*, Δ*PeGβ* and Δ*PeGγ* mutants. **C** Venn diagram of DEGs in Δ*PeGαⅠ*, Δ*PeGβ* and Δ*PeGγ* mutants. **D** GO classification of DEGs in Δ*PeGαⅠ* and shared DEGs in Δ*PeGβ* and Δ*PeGγ*. **E** Heatmap of gene expression in polarized growth pathways in WT and deletion mutants. Red, green, and gray indicate up-regulation, down-regulation, and no change, respectively. **F** RT-qPCR validation of DEGs involved in polarized growth pathways. Primers are listed in Table S3. *β-tubulin* served as the internal control. Data represent mean ± SEM (*n* = 3)

Transcriptome sequencing was conducted on WT, Δ*PeGαI*, ∆*PeGβ*, and ∆*PeGγ* strains to investigate the regulatory mechanisms of PeGαI, PeGβ, and PeGγ in hyphal polarity growth and pathogenicity (Fig. 6A). Comparative analysis identified 2065, 1548, and 1303 differentially expressed genes (DEGs) in Δ*PeGαI*, ∆*PeGβ*, and ∆*PeGγ*, respectively, with a predominance of upregulated genes (Fig. 6B). Notably, ∆*PeGβ* and ∆*PeGγ* shared 1109 DEGs, accounting for 71.64% and 85.11% of their total DEGs, respectively, suggesting synergistic regulatory roles between PeGβ and PeGγ (Fig. 6C). Gene ontology (GO) enrichment analysis revealed that DEGs from all three mutants were enriched in biological processes and cellular components related to fungal polarity growth, including cell wall/membrane/envelope biosynthesis, signal transduction mechanisms, intracellular trafficking, secretion, vesicle transport, and cytoskeleton (Fig. 6D). Further analysis highlighted pathways critical for hyphal polarity growth, such as small GTPase regulation, vesicle transport/cell wall dynamics, microfilament/microtubule cytoskeletal regulation, and calcium/calcineurin signaling (Fig. 6E, Table S2). The expression patterns of DEGs in ∆*PeGβ* and ∆*PeGγ* were highly consistent but distinct from those in Δ*PeGαI*, suggesting collaborative regulation by PeGβ and PeGγ, while PeGαI exhibited a distinct regulatory role. RT-qPCR validation confirmed that the expression patterns of eight key genes in these pathways were consistent with the RNA-seq results (Fig. 6F). These results collectively indicated that the absence of *PeGαI*, *PeGβ*, and *PeGγ* disrupts the expression of genes involved in hyphal polarity growth pathways, potentially affecting cytoskeleton organization, membrane stability, and cell division, ultimately resulting in abnormal hyphal growth and branching patterns.

### PeGαⅠ and PeGαⅢ are crucial for activating the cAMP-PKA signaling pathway

**Fig. 7:**
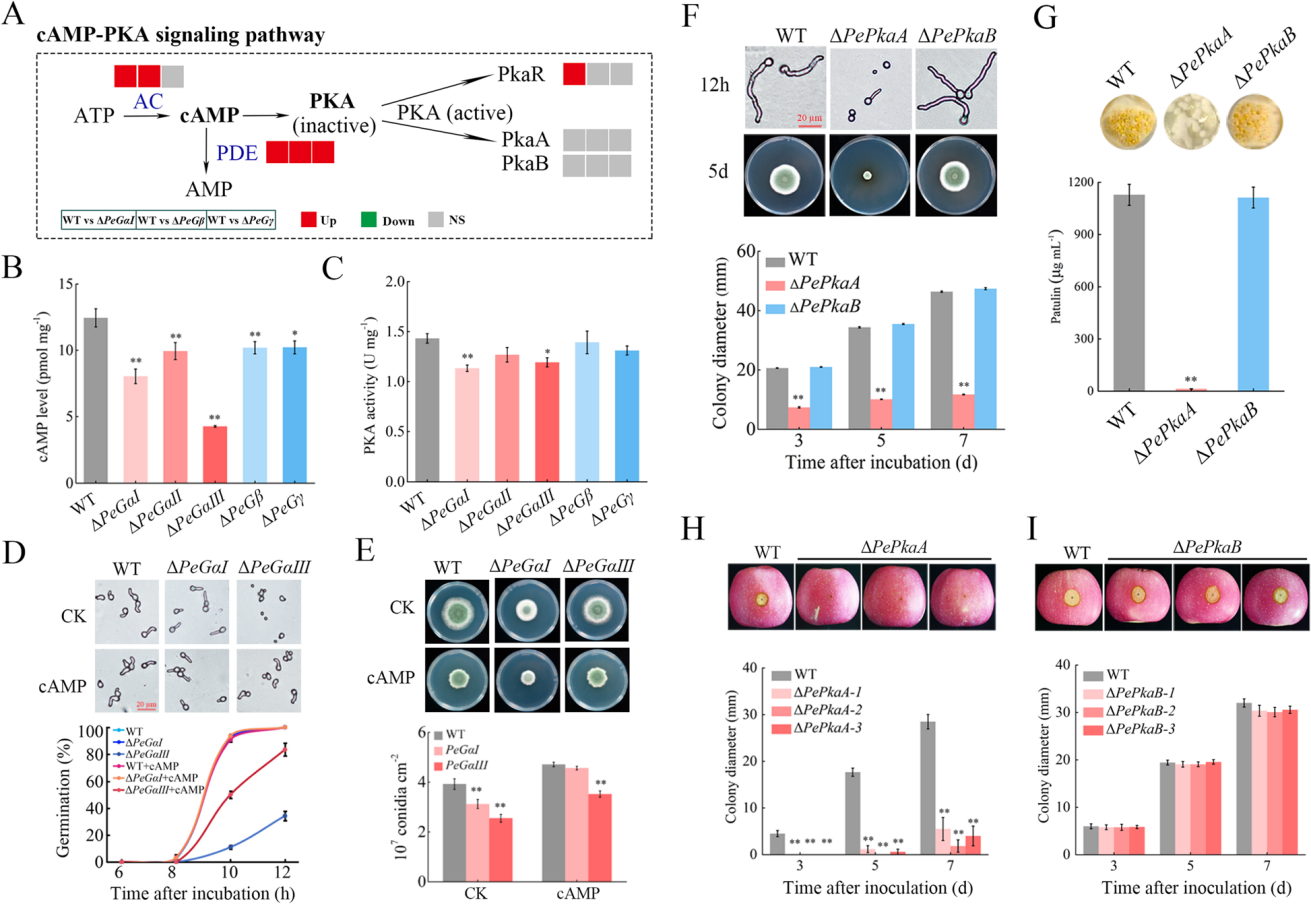
PeGαⅠ and PeGαⅢ are crucial for activating the cAMP-PKA signaling pathway in *P. expansum*. **A**. Expression patterns of DEGs in the cAMP-PKA pathway in Δ*PeGαⅠ*, Δ*PeGβ* and Δ*PeGγ* mutants. **B**, **C** Intracellular cAMP levels (**B**) and PKA activity (**C**) in the WT and deletion mutants of G protein genes. **D**, **E** Conidial germination (**D**) and conidiation (**E**) of WT, Δ*PeGαⅠ*, and Δ*PeGαⅢ* strains with or without 2 mM cAMP. **F** Growth of WT, Δ*PePkaA*, and Δ*PePkaB* strains on PDA at 25 °C for 7 d. **G** Patulin production by WT, Δ*PePkaA*, and Δ*PePkaB* strains in vitro. **H**, **I** Pathogenicity of WT, Δ*PePkaA*, and Δ*PePkaB* strains on apple fruit. Lesion diameters were measured at 3, 5, and 7 dpi. Data represent mean ± SEM (*n* = 3). *P < 0.05, **P < 0.01

The cAMP-PKA signaling pathway is a well-established regulatory pathway mediated by G proteins. Transcriptome analysis demonstrated that G protein subunits modulate the expression of certain genes within this pathway (Fig. 7A). To further elucidate the regulatory role of G protein subunits in the cAMP-PKA pathway, the cAMP levels and PKA activity were quantified in WT and G protein mutant strains. The results revealed a significant reduction in cAMP levels in all G protein mutants compared to WT, with notable decreases of 35.38% and 65.76% in Δ*PeGαI* and Δ*PeGαIII*, respectively (Fig. 7B). Correspondingly, PKA activity was markedly lower in Δ*PeGαI* and Δ*PeGαIII* (Fig. 7C). Furthermore, exogenous supplementation with 2 mM cAMP restored spore germination in Δ*PeGαIII* and increased sporulation in both mutants (Fig. 7D, E). These results highlight the critical roles of PeGαI and PeGαIII in regulating the cAMP-PKA signaling pathway in *P. expansum*.

Functional analysis of the key genes in the pathway, *PePkaA* and *PePkaB*, demonstrated that Δ*PePkaA* exhibited inhibited hyphal growth, reduced pathogenicity, and decreased patulin production, resembling the phenotypes of Δ*PeGαI* and Δ*PeGαIII*, whereas Δ*PePkaB* showed no significant differences compared to WT (Fig. S5; Fig. 7F-I). Overall, these findings indicate that PeGαI and PeGαIII modulate the cAMP-PKA signaling pathway, thereby impacting the pathogenicity and patulin production of *P. expansum*.

### PeGβ and PeGγ modulate the MAPK signaling pathway

**Fig. 8:**
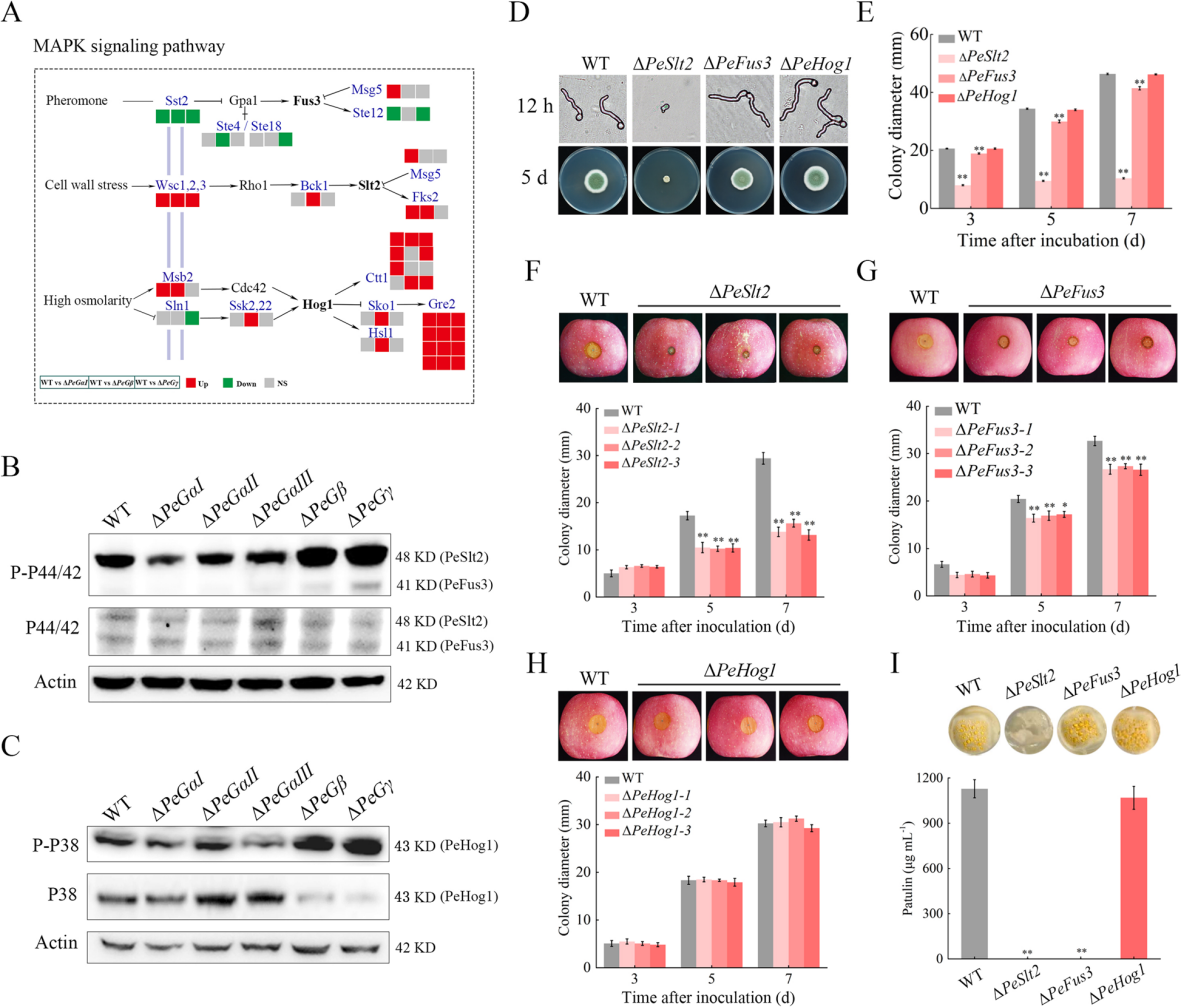
PeGβ and PeGγ modulate the MAPK signaling pathway in *P. expansum*. A Expression patterns of DEGs in the MAPK pathway in Δ*PeGαⅠ*, Δ*PeGβ* and Δ*PeGγ* mutants. **B** Expression and phosphorylation of Fus3 (41 kDa) and Slt2 (48 kDa) in the WT and deletion mutants. Western blots were probed with anti-P44/42, anti-P-P44/42, and anti-actin antibodies. **C** Expression and phosphorylation of Hog1 (43 kDa) in the WT and deletion mutants. Western blots were probed with anti-P38, anti-P-P38, and anti-actin antibodies. **D**, **E** Growth of WT, Δ*PeSlt2*, Δ*PeFus3*, and Δ*PeHog1* strains on PDA at 25 °C for 7 d. **F**–**H** Pathogenicity of WT, Δ*PeSlt2*, Δ*PeFus3*, and Δ*PeHog1* strains on apple fruit. Lesion diameters were measured at 3, 5, and 7 dpi. **I** Patulin production by WT, Δ*PeSlt2*, Δ*PeFus3*, and Δ*PeHog1* strains in vitro. Data represent mean ± SEM (*n* = 3). **P < 0.01

The MAPK signaling pathway is another critical regulatory pathway mediated by G proteins. Transcriptome analysis revealed that the expression of multiple genes within this pathway is influenced by G protein subunits (Fig. 8A). Phosphorylation levels of core MAP kinases (PeSlt2, PeFus3, and PeHog1) were measured in WT and G protein mutant strains. The results revealed a significant increase in PeSlt2 and PeFus3 phosphorylation levels in Δ*PeGβ* and Δ*PeGγ* mutants compared to the WT, with minimal changes observed in other mutants, such as a slight reduction in Δ*PeGαI* (Fig. 8B). Moreover, PeHog1 phosphorylation levels were higher in Δ*PeGβ* and Δ*PeGγ* mutants than in WT (Fig. 8C), suggesting that PeGβ and PeGγ function as negative regulators in the MAPK signaling pathway.

Genetic analysis of MAPK pathway components revealed that Δ*PeSlt2* and Δ*PeFus3* exhibited phenotypes similar to those of Δ*PeGβ* and Δ*PeGγ*, including inhibited hyphal growth, reduced pathogenicity, and diminished patulin production (Fig. S5; Fig. 8D-I). In contrast, Δ*PeHog1* showed no significant differences from WT. These findings emphasize the importance of PeSlt2 and PeFus3 in growth, pathogenicity, and patulin biosynthesis, with PeSlt2 playing a central role. Collectively, the results suggest that PeGβ and PeGγ regulate the MAPK signaling pathway, thereby influencing the pathogenicity and patulin production of *P. expansum*.

## Discussion

*P. expansum* is a major postharvest pathogen that causes fruit decay and patulin contamination, leading to economic losses (Li et al. 2020a; Luciano-Rosario et al. 2020). Understanding the molecular mechanisms of pathogenicity and patulin biosynthesis in *P. expansum* is essential for developing effective control strategies (Zhang et al. 2021a, b). The information gathered in this work suggests that G-protein signaling pathways act as key regulators of both pathogenicity and patulin biosynthesis in *P. expansum*. Deletion of *PeGαI*, *PeGβ*, and *PeGγ* resulted in reduced virulence in fruit hosts, along with decreased patulin biosynthesis. Notably, these mutants exhibit straight hyphal growth, abnormal branching, potentially limiting nutrient acquisition and impacting vitality and pathogenicity. Further investigations revealed that PeGαI is a regulator of conidiation, virulence, and patulin production through the cAMP-PKA signaling pathway, while PeGβ and PeGγ are involved in fungal development and pathogenicity through MAPK-mediated phosphorylation cascades. These findings highlight the critical roles of PeGαI, PeGβ, and PeGγ in regulating pathogenicity and mycotoxin production through multiple signaling pathways in *P. expansum*.

The filamentous morphology of pathogenic fungi is crucial for successful host cell invasion. Filamentous fungi, as highly polarized eukaryotic cells, achieve indeterminate radial growth through continuous elongation and optimize nutrient acquisition via secondary branching (Takeshita 2016; Schmieder et al. 2019). In *P. expansum*, the WT strain exhibits a characteristic wave-like growth pattern, whereas the ∆*PeGαI*, ∆*PeGβ*, and ∆*PeGγ* mutants display straight hyphal growth with significantly reduced pathogenicity in fruit (Figs. 3; 4C). Similar phenotypes have been observed in other pathogenic fungi, such as *Cochliobolus heterostrophus* and *Verticillium dahliae*, where *Gβ* mutants exhibit straight hyphal growth and decreased virulence in maize, tomato, and eggplant (Ganem et al. 2004; Tzima et al. 2012). These findings highlight the critical role of G protein-mediated signaling pathways in maintaining fungal hyphal morphology.

Hyphal branching, which includes both apical and lateral patterns, is crucial for mycelial colony development and fungal interactions with hosts (Harris 2008). In *P. expansum*, the WT strain forms both apical and lateral branches, creating a uniform, radially distributed network (Fig. 4C). In contrast, the ∆*PeGαI* mutant produces only lateral branches, resulting in an uneven hyphal distribution and an irregular prism-like morphology (Fig. 4C). The ∆*PeGβ* and ∆*PeGγ* mutants exhibit a significant reduction in both branching types, with apical branches predominating, leading to an irregular broom-like structure (Fig. 4C). Similarly, the Gβ subunit mutant Δ*fgb1* in *Fusarium oxysporum* displays abnormal hyphal growth, characterized by reduced apical branching and decreased pathogenicity in tomatoes (Delgado-Jarana et al. 2005). Based on these observations, we hypothesize that the aberrant growth patterns of G protein subunit mutants may impair their ability to efficiently acquire nutrients from the surrounding environment, thereby reducing fungal vitality and pathogenicity. This hypothesis is supported by competitive subculture experiments, which reveal that these mutants lack a competitive advantage and exhibit a significant decline in viability during subculturing.

Transcriptomic sequencing and comparative analysis were conducted on the WT and Δ*PeGαI*, Δ*PeGβ*, and Δ*PeGγ* mutants to investigate the mechanisms of abnormal hyphal growth in the G protein mutants. The study identified DEGs enriched in pathways related to fungal polar growth, including small GTPase signaling, vesicle trafficking, microfilament and microtubule dynamics, and calcineurin signaling (Fig. 6E, Table S2). The GTPase module is necessary for bud-site recognition in both axial and bipolar growth patterns in filamentous fungi (Cullen and Sprague 2012). Our findings suggest that a majority of DEGs within the GTPase module were upregulated in the Δ*PeGαI*, Δ*PeGβ*, and Δ*PeGγ* mutants, potentially disrupting cellular polarity and altering hyphal growth patterns. Microtubules, which are essential for vesicular transport pathways, are critical for maintaining hyphal growth direction (Riquelme et al. 2018). In the mutants, we observed significant downregulation of key genes related to microfilament and microtubule dynamics, such as *tubulin*, *kinesin*, *Arp2/3 complex*, and *profilin*. This transcriptional alteration may impair microtubule dynamics, resulting in restricted hyphal growth and reduced morphological flexibility. Furthermore, the hyphal tip Ca^2^⁺ gradient, which regulates hyphal growth and morphogenesis by modulating the apical actin network (Kim et al. 2012), was affected similarly. In line with the known inhibitory impact of a tip-high Ca^2^⁺ gradient on fungal branching, as demonstrated in *N. crassa* (Harris 2019), a notable upregulation of calcium signaling-related genes was observed in the G protein mutants of *P. expansum*. This upregulation is speculated to impede hyphal branching by disrupting tip synthesis processes. Notably, the expression patterns of DEGs in Δ*PeGβ* and Δ*PeGγ* were highly consistent across polar growth-related pathways, whereas Δ*PeGαI* exhibited distinct transcriptional profiles. These findings suggest that PeGβ and PeGγ function synergistically to regulate hyphal polar growth in *P. expansum*, while PeGαI operates through a unique regulatory mechanism. However, the specific mechanisms by which PeGαI, PeGβ, and PeGγ differentially regulate polar growth in *P. expansum* require further investigation.

In addition to their roles in polar growth, PeGαⅠ and PeGαⅢ are crucial for cAMP-PKA signaling, whereas PeGβ and PeGγ regulate MAPK signaling. The differential phosphorylation patterns of G protein subunits may originate from their structural specializations. GαI and GαIII possess N-terminal myristoylation sites that could direct them to membrane microdomains rich in adenylate cyclase (Song et al. 1997), potentially enhancing localized cAMP-PKA pathway activation. In mammals, Gsα (homolog of GαIII) directly binds to adenylyl cyclase (Tesmer et al. 1997), leading to the generation of cAMP by activated adenylate cyclase. This activation triggers PKA activation, which subsequently regulates cellular responses through phosphorylation of downstream targets and modulation of their expression (Sun et al. 2022). In *Saccharomyces cerevisiae*, Gpa2 (homolog of GαI) participates in fungal development by modulating cAMP signaling (Kubler et al. 1997). In filamentous fungi, mutations in homologous genes of *GαI* and *GαIII* can alter intracellular cAMP levels, with supplementation of exogenous cAMP often restoring observed defects in these mutants (Song et al. 2017; Hu et al. 2018b; Liu et al. 2018), suggesting that Gα proteins mediate signal transfer to the cAMP pathway. The Gβ subunit’s WD40 repeat domain forms a circular β-propeller structure that directly interacts with MAPK scaffolding proteins, enabling regulation of the MAPK cascade. This is evolutionarily conserved, as demonstrated in *S. cerevisiae*, where the Gβγ dimer binds scaffold protein Ste5 and PAK kinase Ste20 to activate the Ste11-Ste7-Fus3/Kss1 phosphorylation cascade (Winters et al. 2005). Genetic analyses in filamentous fungi indicate that Gβ proteins can transmit signals to the MAPK pathway, similar to the *S. cerevisiae* pheromone response cascade, as observed in *Cryptococcus neoformans* (Wang et al. 2000) and *Magnaporthe grisea* (Nishimura et al. 2003). Additionally, certain Gβ-like proteins, characterized by a seven-bladed β-propeller structure resembling the Gβ subunit, are implicated in regulating the MAPK signaling pathway. These proteins have been shown to directly interact with specific MAPK cascade proteins in fungi, such as Asc1-Ste20 in *S. cerevisiae* (Zeller et al. 2007), MoMip11-Mck1/Ste50 in *Magnaporthe oryzae* (Li et al. 2017), and Bcgbl1-BcSte50 in *B. cinerea* (Tang et al. 2023).

The differential pathogenicity among G protein subunit mutants arises from their distinct roles in regulating key virulence determinants. PeGαI and PeGαIII primarily govern the cAMP-PKA pathway (Fig. 7), while PeGβ and PeGγ negatively regulate MAPK signaling (Fig. 8). Both pathways are essential for fungal pathogenicity. Specifically, the cAMP-PKA pathway governs stress response, metabolism, and infection structure development (Fuller and Rhodes 2012), whereas the MAPK pathway is essential for preserving cell wall integrity and enabling host penetration (Zhang et al. 2021a, b). The functional importance of these pathways is further supported by genetic evidence showing that the catalytic subunit PePkaA in cAMP-PKA pathway and the MAP kinases PeSlt2 and PeFus3 in MAPK pathway are essential for pathogenicity (Figs. 7G; 8F, G). On the other hand, PeGαI, PeGβ, and PeGγ subunits regulate hyphal polarity through coordinated regulation of small GTPase signaling, cytoskeletal dynamics, and vesicle trafficking (Fig. 6E, Table S2). Disruption of these subunits leads to severe morphological defects. Δ*PeGαI* exhibits irregular rhomboid growth with only lateral branching, while Δ*PeGβ/γ* mutants display a broom-like morphology with apical branching dominance (Fig. 4C). These structural abnormalities may impair host tissue penetration and reduce nutrient acquisition efficiency, ultimately compromising pathogenic success. In contrast, Δ*PeGαII* shows minimal pathogenicity reduction due to its limited impact on these core pathways and hyphal morphology. Δ*PeGαIII* displays a lesser degree of pathogenicity reduction compared to Δ*PeGαI*, Δ*PeGβ*, and Δ*PeGγ* mutants, as it maintains relatively normal hyphal morphology despite its cAMP-PKA defects. This differential pathogenicity regulation observed here reflects the conserved G protein functions found in other phytopathogens like *B. cinerea* (Gronover et al. 2001; Döhlemann et al. 2006; Tang et al. 2021) and *M. grisea* (Liu and Dean 1997; Nishimura et al. 2003; Li et al. 2015b), where GαI/Gβ/Gγ subunits consistently show stronger phenotypic effects than other family members.

G protein-mediated signaling pathways are integral not only to fungal growth and pathogenicity but also to the regulation of biosynthesis for various mycotoxins. Previous research has demonstrated that G proteins positively regulate aflatoxin in *A. flavus* (Liu et al. 2018), gliotoxin in *A. fumigatus* (Choi et al. 2020), and melanin and HT toxin in *S. turcica* (Li et al. 2018). Conversely, they exhibit negative regulation of mycotoxins such as deoxynivalenol and zearalenone in *G. zeae* (Yu et al. 2008). However, the specific regulatory mechanisms remain unclear. Recently, Zhang et al. (2024) revealed that the deletion of *Gα3* significantly reduced patulin content in *P. expansum*, accompanied by decreased expression levels of most genes within the patulin biosynthesis cluster. In the present study, deletions of *PeGαI*, *PeGαIII*, *PeGβ*, and *PeGγ* all led to a marked reduction in patulin biosynthesis both in vivo and in vitro (Fig. 5A, B). Gene expression analysis showed that, except for *PatO*, 14 other genes were significantly downregulated in ∆*PeGβ* and ∆*PeGγ* mutants (Fig. 5C), indicating that PeGβ and PeGγ influence patulin biosynthesis by regulating the expression of the gene cluster. In contrast, only a few genes exhibited significant downregulation in ∆*PeGαI* and ∆*PeGαIII* mutants (Fig. 5C). This regulatory pattern resembles that observed for the sulfur metabolism regulator PeMetR (Chen et al. 2021) and the carbon metabolism regulator CreA (Tannous et al. 2018) in *P. expansum*, where gene deletion mutants fail to produce patulin, yet most genes in the biosynthesis cluster remain normally expressed. Additionally, it has been demonstrated that this regulatory pattern is attributed to the lack of metabolic precursor substances due to the absence of regulatory factors in *A. nidulans* (Zhang and Keller 2004), which may explain the reduced patulin production in ∆*PeGαI* and ∆*PeGαIII* mutants.

**Fig. 9:**
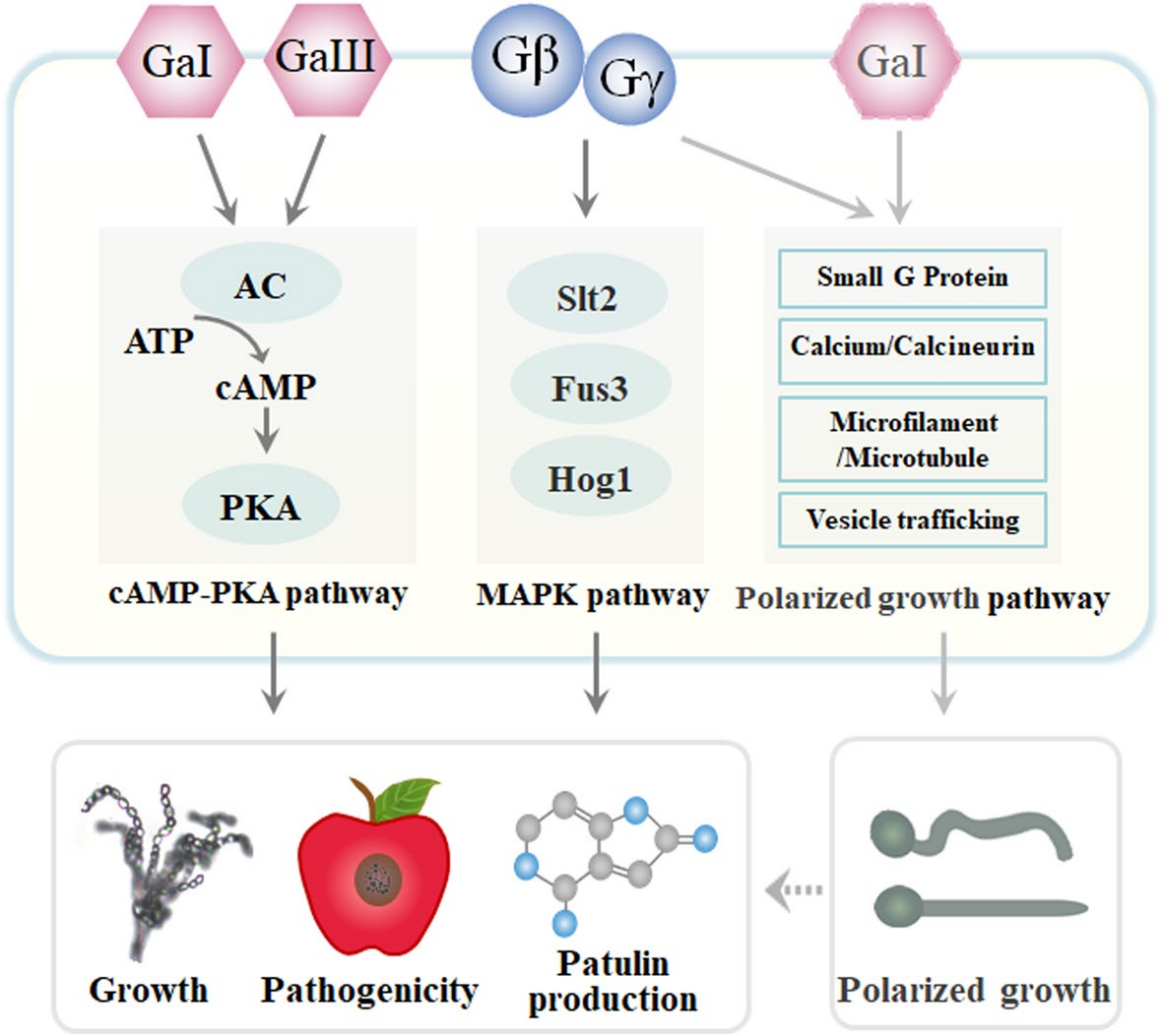
Proposed model for G-protein signaling pathways regulating the growth, pathogenicity and patulin biosynthesis in *P. expansum*. G protein subunits regulate hyphal growth, virulence, and patulin production in *P. expansum* through multiple pathways. PeGαI and PeGαIII primarily function via the cAMP-PKA pathway, while PeGβ and PeGγ modulate the MAPK pathway, influencing virulence and patulin production. Additionally, PeGαI, PeGβ, and PeGγ impact hyphal polarity and vitality, which may further affect growth and pathogenicity

In summary, this study elucidates the regulatory roles of five G protein subunits in *P. expansum*, demonstrating their differential effects on hyphal growth, virulence, and patulin biosynthesis through distinct signaling cascades (Fig. 9). PeGαⅠ and PeGαⅢ primarily regulate cAMP-PKA signaling, while PeGβ and PeGγ modulate MAPK pathways. Furthermore, PeGαⅠ, PeGβ, and PeGγ have broad effects on fungal hyphal polarity, resulting in reduced vitality that may impact growth and pathogenicity. These findings offer new insights into the molecular mechanisms of *P. expansum* infection, identifying potential targets for controlling blue mold and mycotoxin contamination in fruits.

## Materials and methods

### Fungal strains and culture conditions

The WT strain *P. expansum* T01, originally isolated from naturally infected apple fruit, served as the parental strain for generating gene deletion mutants (Li et al. 2015a). All strains were cultured on potato dextrose agar (PDA) plates at 25 °C under dark conditions, and conidia were collected at 7 dpi for further experiments.

### Sequence and cluster analysis of G protein subunits

Homologs of *N. crassa* G proteins (GNA-1 (XP_957133), GNA-2 (Q05424), GNA-3 (XP_962205), GNB-1 (AAM53552), GNG-1 (AAV83542.1)) in *P. expansum* were identified using BLASTp, including PeGαI (XP_016596079.1), PeGαII (XP_016601136.1), PeGαIII (XP_016596161.1), PeGβ (XP_016601560.1), and PeGγ (XP_016595165.1). G protein sequences from other species were retrieved from published literature (Li et al. 2007) and the NCBI database (http://www.ncbi.nlm.nih.gov/). Multiple sequence alignment was performed using Clustal W, and a phylogenetic tree was constructed using the Neighbor-Joining (NJ) method in MEGA 6.0 software with 1000 bootstrap replicates. Conserved protein domains were analyzed using the SMART database (http://smart.embl-heidelberg.de).

### Construction of gene knockout and complementary strains

Gene knockout mutants were constructed using the homologous recombination strategy and the *Agrobacterium tumefaciens*-mediated transformation (ATMT) method as described by Li et al. (2015a). Flanking regions (approximately 1 kb) of the target genes were cloned and integrated upstream or downstream of the hygromycin B resistance cassette (*hph*) in the pCHPH vector to construct knockout vectors. Transformants were selected with 250 μg mL^−1^ hygromycin B, and positive ones were confirmed by PCR and Southern blot analysis. For gene complementation, the full-length target genes were inserted into the pCNEO vector with a neomycin resistance (*neo*) gene. Transformants were screened with 250 μg mL^−1^ G418 and validated by PCR. Details of all primers used for gene knockout and complementation can be found in Table S1 and S5.

### Subcellular localization of G protein subunits

The G protein subunit localizations were determined following the method outlined by Li et al. (2019). The coding sequences of the G protein genes were amplified using gG proteins-F/R primers (Table S1). Fusion expression vectors of the target genes and *egfp* were generated and subsequently transformed into WT using the ATMT method. Transformants were selected using 250 μg mL^−1^ hygromycin B. Spores of PeGαI::eGFP, PeGαII::eGFP, PeGαIII::eGFP, PeGβ::eGFP, and PeGγ::eGFP were cultured in CY media at 25 °C for 13–15 h. Fluorescent signals were visualized using a confocal Zeiss 980 laser scanning microscope (Zeiss, Oberkochen, Germany). FM4-64 (Thermo Fisher Scientific, Waltham, MA, USA) was used to label the plasma membrane following the manufacturer’s instructions.

### Phenotype analysis

Phenotypic analysis was performed following established protocols (Chen et al. 2021, 2023; Xu et al. 2024), with all experiments conducted in triplicate.

(i) Mycelial growth
  For growth assays, 5 μL of conidial suspension (1 × 10^5^ conidia mL^−1^) was inoculated onto PDA and Czapek yeast agar (CYA) media and incubated at 25 °C in the dark. Colony diameters were measured and photographed at 3, 5, and 7 dpi.
(ii) Conidiation
  Conidia were harvested by flooding PDA plates with 0.05% Tween 20 at 3, 5, and 7 dpi. The suspension was filtered through sterile gauze, and conidial counts were determined using an automated cell counter (IY1200; Countstar). For cAMP supplementation assays, 5 μL of conidial suspension (1 × 10^5^ conidia mL⁻^1^) was inoculated onto PDA plates supplemented with 0 or 2 mM cAMP (S18050, Shanghai Yuanye Bio-Technology Co., Ltd), and conidial counts were recorded at 7 dpi.
(iii) Conidial germination
  In vitro germination assays: Conidial suspensions (2 × 10⁷ conidia mL⁻^1^) were evenly spread on PDA media overlaid with cellophane sheets. Germination rates were assessed using a microscope (Leica DM2000, Germany) after 6–20 h of incubation. In vivo germination assays: Apple fruits were wounded to create 3 cm × 3 cm lesions, and conidial suspensions (2 × 10⁷ conidia mL⁻^1^) were applied to the wound surfaces. Thin surface slices were examined under a microscope after 8–12 h of incubation. For cAMP supplementation assays, conidial suspensions were spread on PDA media supplemented with 0 or 2 mM cAMP and observed under a microscope.
(iv) Pathogenicity
  Pathogenicity was assessed using apple and pear fruits. Four equidistant wounds were made on the equator of each fruit using a sterile nail, and 5 μL of conidial suspension (1 × 10^5^ conidia mL⁻^1^) was inoculated into each wound. Inoculated fruits were incubated at 25 °C for 7 d, and lesion diameters were measured and photographed at 3, 5, and 7 dpi. Each strain was tested on at least eight fruits.
(v) Patulin biosynthesis
  To assess patulin biosynthesis, 1 μL of conidial suspension (1 × 10⁶ conidia mL⁻^1^) was spread on PDA media overlaid with 1 × 1 cm cellophane sheets and incubated at 25 °C for 36 h. Mycelia and cellophane sheets were transferred to 1 mL of CY medium. After 2 d, the medium was filtered through a 0.45 μm membrane for HPLC analysis, and mycelia were collected for RNA extraction and expression analysis of patulin cluster genes. Each strain was tested on six cellophane sheets. Total RNA extraction, reverse transcription, and RT-qPCR were performed as described by Li et al. (2015a). Primers for patulin cluster genes are listed in Table S4.
(vi) Stress tolerance tests
  For light stress assays, 5 μL of conidial suspension (1 × 10^5^ conidia mL⁻^1^) was inoculated onto PDA plates and exposed to continuous white light (7000 lx). For other stress treatments, conidial suspensions were cultured on PDA plates supplemented with 1 M NaCl (osmotic stress), 3 mg mL⁻^1^ Congo red (cell wall stress), or adjusted to pH 3.0 or 8.0 using 0.2 M Na₂HPO₄−0.1 M citric acid buffer (Chen et al. 2018). Plates were incubated at 25 °C for 7 d. Minimal medium (MM) was used as the base for carbon and nitrogen source experiments. Glucose in MM was replaced with 1% sucrose or 1% galactose as alternative carbon sources, while NaNO₃ was replaced with 3 g L⁻^1^ tryptone or 70 mM NH₄Cl as alternative nitrogen sources. Conidial suspensions were inoculated onto the modified media and incubated at 25 °C for 7 d.

### RNA-seq analysis

Conidial suspensions (1 × 10^3^ conidia mL⁻^1^) of the WT, Δ*PeGαI*, Δ*PeGβ*, and Δ*PeGγ* strains were evenly spread on PDA media overlaid with cellophane sheets and incubated at 25 °C for 36 h. Total RNA was extracted from samples collected from three independent biological replicates using the TRIzol method. cDNA library preparation and Illumina sequencing were performed following the protocol described by Li et al. (2020b). DEGs were identified using a threshold of fold change ≥ 1.5 and a false discovery rate (FDR) < 0.01. To validate the RNA-seq results, RT-qPCR was conducted on eight target genes, with primers listed in Table S3.

### Competition experiment

Competition experiments were performed as described by Luciano-Rosario et al. (2022), where Δ*PeGαI* and Δ*PeGβ* strains were individually competed against the WT control. Conidial suspensions (1 × 10^5^ conidia mL⁻^1^) of Δ*PeGαI*, Δ*PeGβ*, WT, and WT::eGFP (Li et al. 2015a) strains were prepared. Spore suspensions of WT::eGFP strain were mixed in equal proportions with those of WT, Δ*PeGαI*, and Δ*PeGβ* strains (P0). Subsequently, 10 µL of each mixed suspension was inoculated onto PDA plates, incubated at 25 °C for 7 d, and observed under a fluorescence microscope. Conidia were harvested by flooding the PDA plates for microscopic analysis and quantification (P1). The same procedure was repeated to obtain P2 samples.

### Assays for MAPK phosphorylation

WT and G protein mutant strains were cultured in CY media at 25 °C for 48 h on a rotary shaker at 180 rpm. Mycelia were collected, flash-frozen in liquid nitrogen, and homogenized in protein lysis buffer (50 mM Tris–HCl, pH 7.5, 150 mM NaCl, 1 mM EDTA, 1% NP-40) supplemented with 1 × protease inhibitor cocktail (C0101, LabLead), phosphatase inhibitor cocktail 2 (P5726, Sigma-Aldrich), and phosphatase inhibitor cocktail 3 (P0044, Sigma-Aldrich), as described by Jiang et al. (2019). The expression and phosphorylation levels of Slt2 and Fus3 were assessed using the p44/42 MAPK (Erk1/2) antibody kit (9102, Cell Signaling Technology) and the Phospho-p44/42 MAPK antibody kit (9101, Cell Signaling Technology), respectively. Similarly, Hog1 expression and phosphorylation were analyzed using the p38 MAPK antibody kit (9212, Cell Signaling Technology) and the Phospho-p38 MAPK antibody kit (4511, Cell Signaling Technology). Anti-β-tubulin (M30109, Abmart) served as the loading control.

### Measurement of cAMP level and PKA activity

Sample preparation for cAMP and PKA activity assays followed the same protocol as for the MAPK phosphorylation assays. For cAMP quantification, 0.1 g of mycelia from WT and G protein mutant strains was homogenized in 0.1 M HCl and centrifuged at 10,000 rpm for 10 min. The supernatant was analyzed using a Direct cAMP ELISA Kit (ADI-900-066A, Enzo Life Sciences) according to the manufacturer’s instructions. PKA activity was measured using the PKA Colorimetric Activity Kit (EIAPKA, Invitrogen, Carlsbad, CA, USA). All experiments were performed in triplicate.

### Statistical analysis

Data are presented as mean ± standard error of the mean (SEM). Statistical analysis was performed using one-way ANOVA in SPSS software (SPSS Inc., Chicago, IL, USA). The Student’s t-test was utilized to assess the significance of differences between two means (*P < 0.05, **P < 0.01).

## Supplementary Information

Supplementary Material 1: Supplemental figures. Fig. S1. Phylogenetic analysis and conserved domain identification of G protein subunits in *P. expansum* and four other fungal species. The phylogenetic tree was constructed using MEGA 6.0 software with the Neighbor-Joining (NJ) method based on amino acid sequence alignments of G protein subunits. Protein IDs for G protein subunits are as follows: PeGαI (XP_016596079.1), PeGαII (XP_016601136.1), PeGαIII (XP_016596161.1), PeGβ (XP_016601560.1), PeGγ (XP_016595165.1); FadA (XP 658255), GanA (XP660694), GanB (XP658620), SfaD (EAA65259), GpgA (EAA63176); GNA-1 (XP957133), GNA-2 (Q05424), GNA-3 (XP962205), GNB-1 (AAM53552), GNG-1 (542AAV83); MAGB (XP368879), MAGC (AAB65427), MAGA (XP363892), MGB1 (BAC01165.1), MGG1 (ABD14415); Gpa1 (AJU16557.1), Gpa2 (AJU51167.1), Ste4p (AJT98994.1), Ste18p (AJR58611.1). Fig. S2. PCR identification of positive transformants for PeGαⅠ::eGFP, PeGαⅡ::eGFP, PeGαⅢ::eGFP, PeGβ::eGFP, and PeGγ::eGFP strains. Fig. S3. Targeted gene disruption and complementation of G protein genes. A. Schematic representation of the gene replacement strategy using a hygromycin B resistance cassette. B. PCR identification of positive transformants in gene deletion strains. C. Confirmation of positive transformants by Southern blot analysis. D. PCR identification of positive transformants in gene complementation strains. Fig. S4. Roles of G protein subunits in stress responses and nutrient sensing in *P. expansum*. A. Colony morphologies of the WT and G protein mutants after 5 d of stress treatments. B. Growth inhibition rate of each strain after 5 d of stress treatments. C. Colony morphologies of the WT and G protein mutants on various carbon and nitrogen sources. D. Growth inhibition rate of each strain after 5 d of incubation. Data are presented as mean ± SEM (*n* = 3). *P < 0.05, **P < 0.01. Fig. S5. PCR identification of positive transformants in gene deletion strains of *PePkaA*, *PePkaB*, *PeSlt2*, *PeFus3*, and *PeHog1* in *P. expansum*.

Supplementary Material 2: Supplemental tables. Table S1. Primer sequences used for manipulation of G protein genes in *P. expansum*. Table S2. List of DEGs involved in polarized growth pathways in Δ*PeGαI*, Δ*PeGβ*, and Δ*PeGγ* strains. Table S3. Primer sequences used for RT-qPCR analysis of DEGs involved in polarized growth pathways. Table S4. Primer sequences used for RT-qPCR analysis of patulin cluster genes. Table S5. Primer sequences used for manipulation of *PeSlt2, PeFus3, PeHog1, PePkaA and PePkaB* in *P. expansum*.

## Data Availability

The data that support the findings of this study are available from the corresponding author upon reasonable request.

## Abbreviations

WT: Wild-type
Gα: G protein alpha subunit
Gβ: G protein beta subunit
Gγ: G protein gamma subunit
GPCRs: G protein-coupled receptors
GDP: Guanosine diphosphate
GTP: Guanosine triphosphate
GTPase: Guanosine triphosphatase
cAMP: Cyclic adenosine monophosphate
PKA: Protein kinase A
MAPK: Mitogen-activated protein kinase
Arp2/3: Actin-related protein 2/3
DEGs: Differentially expressed genes
GO: Gene ontology
RT-qPCR: Reverse transcription-quantitative PCR
HPLC: High-performance liquid chromatography
NCBI: National Center for Biotechnology Information
ATMT: *Agrobacterium tumefaciens*-mediated transformation
GFP: Green fluorescent protein
hpi: Hours post-inoculation
dpi: Days post-inoculation
PDA: Potato dextrose agar
CY: Czapek yeast extract
MM: Minimal medium
