# Supplementary material for: Dissecting G-protein signaling pathways in the fruit pathogen Penicillium expansum: implications for pathogenesis and patulin production
Source: Mol Hortic. 2026 Apr 8;6:28. doi: 10.1186/s43897-025-00211-w (PMC13059215; doi:10.1186/s43897-025-00211-w)
Supplement: Supplementary file 1 — Supplementary Material 1: Supplemental figures. Fig. S1. Phylogenetic analysis and conserved domain identification of G protein subunits in P. expansum and four other fungal species. The phylogenetic tree was constructed using MEGA 6.0 software with the Neighbor-Joining (NJ) method based on amino acid sequence alignments of G protein subunits. Protein IDs for G protein subunits are as follows: PeGαI (XP_016596079.1), PeGαII (XP_016601136.1), PeGαIII (XP_016596161.1), PeGβ (XP_016601560.1), PeGγ (XP_016595165.1); FadA (XP 658255), GanA (XP660694), GanB (XP658620), SfaD (EAA65259), GpgA (EAA63176); GNA-1 (XP957133), GNA-2 (Q05424), GNA-3 (XP962205), GNB-1 (AAM53552), GNG-1 (542AAV83); MAGB (XP368879), MAGC (AAB65427), MAGA (XP363892), MGB1 (BAC01165.1), MGG1 (ABD14415); Gpa1 (AJU16557.1), Gpa2 (AJU51167.1), Ste4p (AJT98994.1), Ste18p (AJR58611.1). Fig. S2. PCR identification of positive transformants for PeGαⅠ::eGFP, PeGαⅡ::eGFP, PeGαⅢ::eGFP, PeGβ::eGFP, and PeGγ::eGFP strains. Fig. S3. Targeted gene disruption and complementation of G protein genes. A. Schematic representation of the gene replacement strategy using a hygromycin B resistance cassette. B. PCR identification of positive transformants in gene deletion strains. C. Confirmation of positive transformants by Southern blot analysis. D. PCR identification of positive transformants in gene complementation strains. Fig. S4. Roles of G protein subunits in stress responses and nutrient sensing in P. expansum. A. Colony morphologies of the WT and G protein mutants after 5 d of stress treatments. B. Growth inhibition rate of each strain after 5 d of stress treatments. C. Colony morphologies of the WT and G protein mutants on various carbon and nitrogen sources. D. Growth inhibition rate of each strain after 5 d of incubation. Data are presented as mean ± SEM (n = 3). *P < 0.05, **P < 0.01. Fig. S5. PCR identification of positive transformants in gene deletion strains of PePkaA, PePkaB, PeSlt2, PeFus3, and PeHog1 in [file 43897_2025_211_MOESM1_ESM.docx]

**
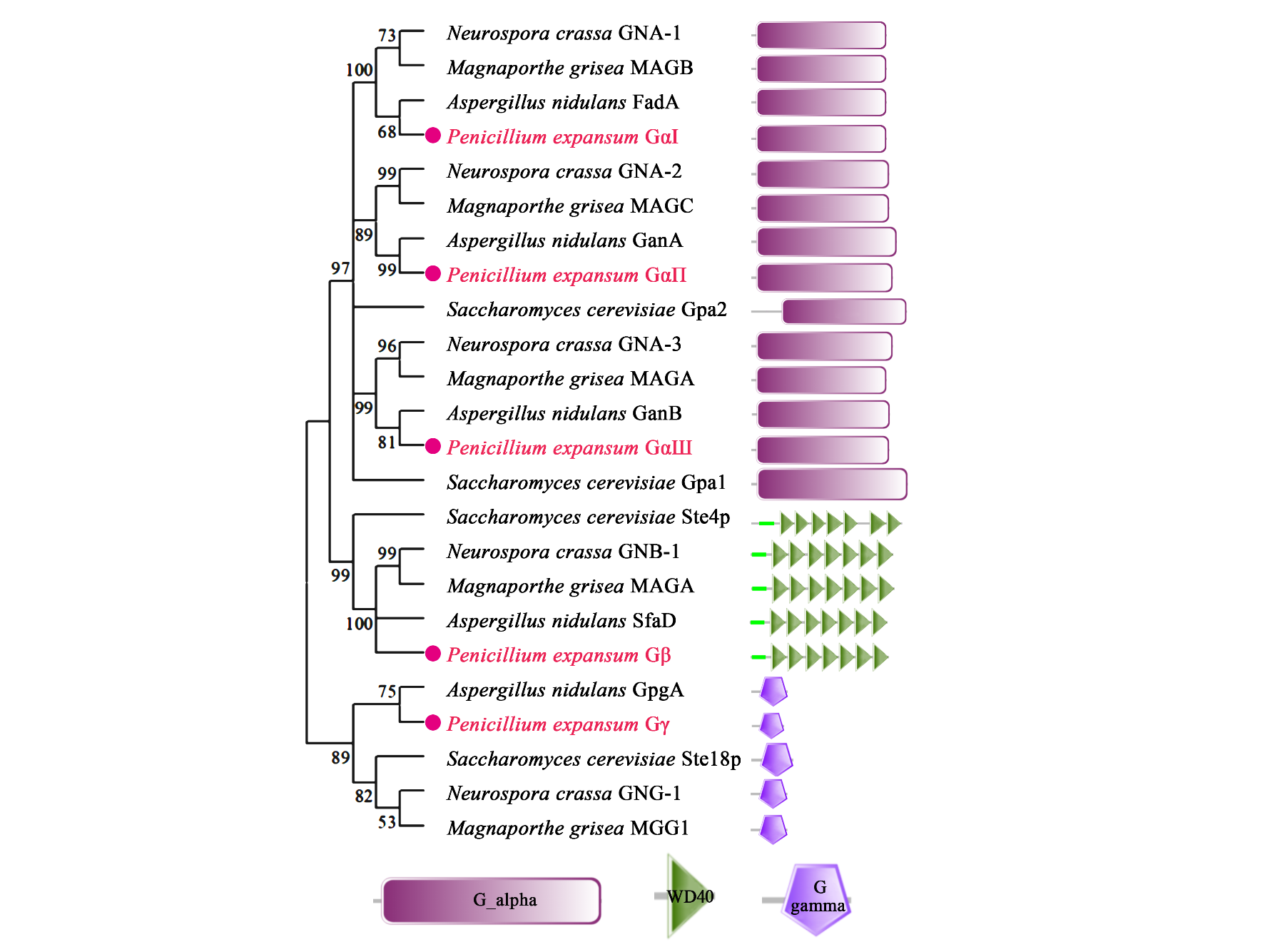
**

**Fig. S1. Phylogenetic analysis and conserved domain identification of G protein subunits in *P. expansum* and four other fungal species.** The phylogenetic tree was constructed using MEGA 6.0 software with the Neighbor-Joining (NJ) method based on amino acid sequence alignments of G protein subunits. Protein IDs for G protein subunits are as follows: PeGαI (XP_016596079.1), PeGαII (XP_016601136.1), PeGαIII ([XP_016596161.1](https://www.ncbi.nlm.nih.gov/protein/XP_016596161.1?report=genbank&log$=prottop&blast_rank=1&RID=U8MWM4WR013)), PeGβ (XP_016601560.1), PeGγ ([XP_016595165.1](https://www.ncbi.nlm.nih.gov/protein/XP_016595165.1?report=genbank&log$=prottop&blast_rank=2&RID=UARK4WZ7016)); FadA (XP 658255), GanA (XP660694), GanB (XP658620), SfaD (EAA65259), GpgA (EAA63176); GNA-1 (XP957133), GNA-2 (Q05424), GNA-3 (XP962205), GNB-1 (AAM53552), GNG-1 (542AAV83); MAGB (XP368879), MAGC (AAB65427), MAGA (XP363892), MGB1 (BAC01165.1), MGG1 (ABD14415); Gpa1 (AJU16557.1), Gpa2 (AJU51167.1), Ste4p (AJT98994.1), Ste18p (AJR58611.1).

**
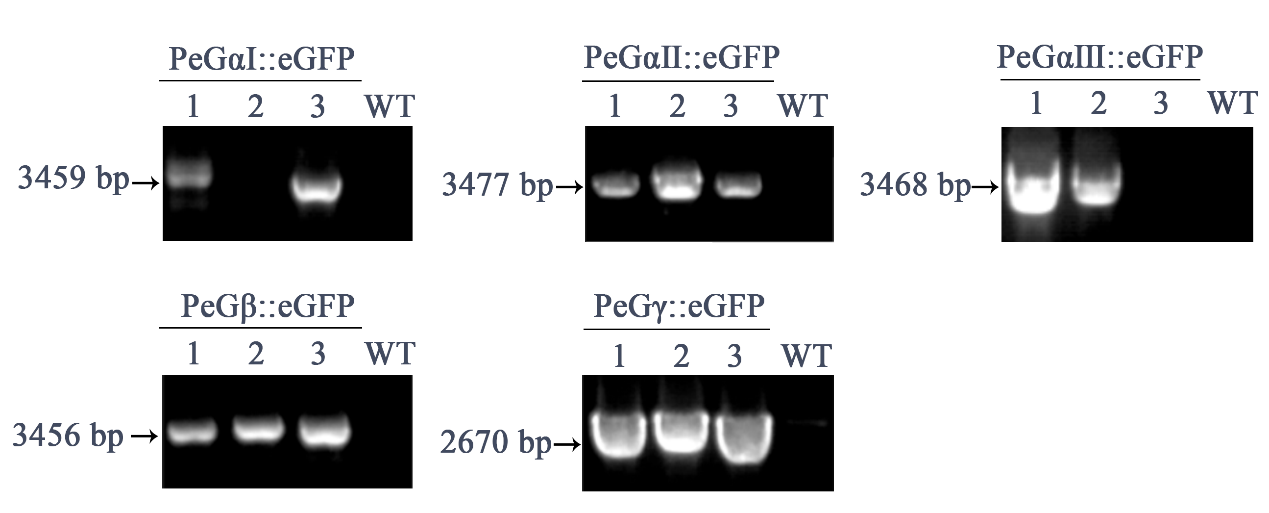
**

**Fig. S2. PCR identification of positive transformants for PeGαⅠ::eGFP, PeGαⅡ::eGFP, PeGαⅢ::eGFP, PeGβ::eGFP, and PeGγ::eGFP strains.**

**
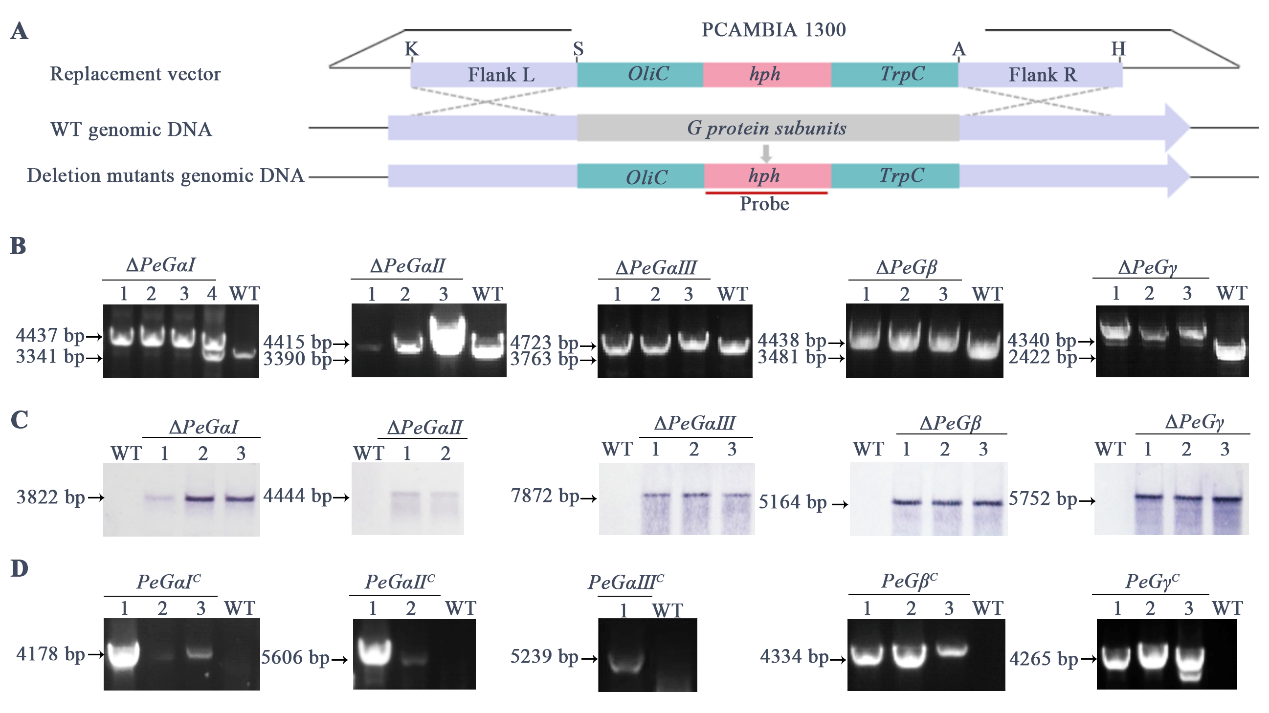
**

**Fig. S3. Targeted gene disruption and** **complementation of G protein genes.** **A**. Schematic representation of the gene replacement strategy using a hygromycin B resistance cassette. **B**. PCR identification of positive transformants in gene deletion strains. **C**. Confirmation of positive transformants by Southern blot analysis. **D**. PCR identification of positive transformants in gene complementation strains.


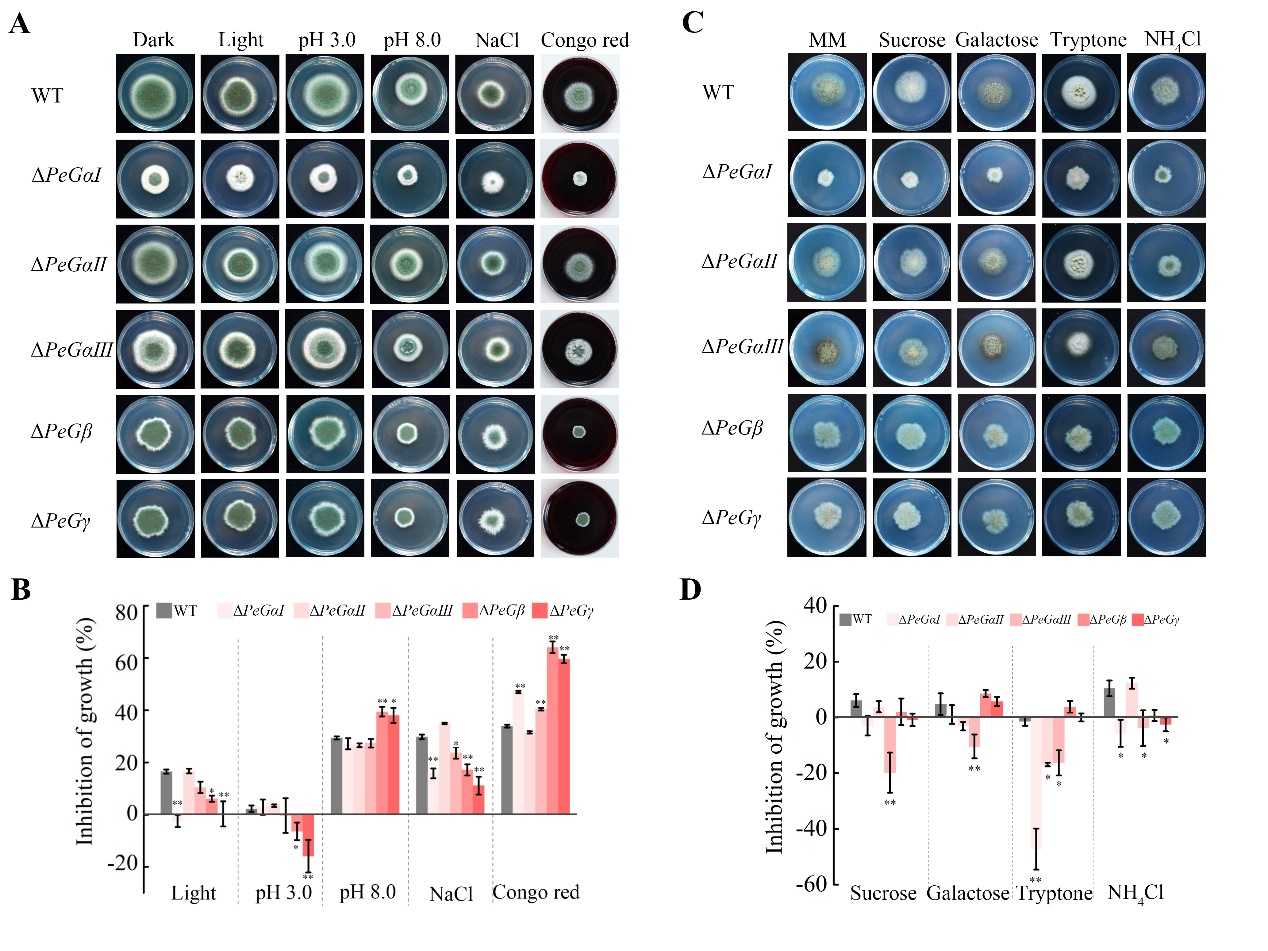


**Fig. S4. Roles of G protein subunits in stress responses and nutrient sensing in *P. expansum*.** **A**. Colony morphologies of the WT and G protein mutants after 5 d of stress treatments. **B**. Growth inhibition rate of each strain after 5 d of stress treatments. **C**. Colony morphologies of the WT and G protein mutants on various carbon and nitrogen sources. **D**. Growth inhibition rate of each strain after 5 d of incubation. Data are presented as mean ± SEM (n = 3). **P* < 0.05, ***P* < 0.01.

**
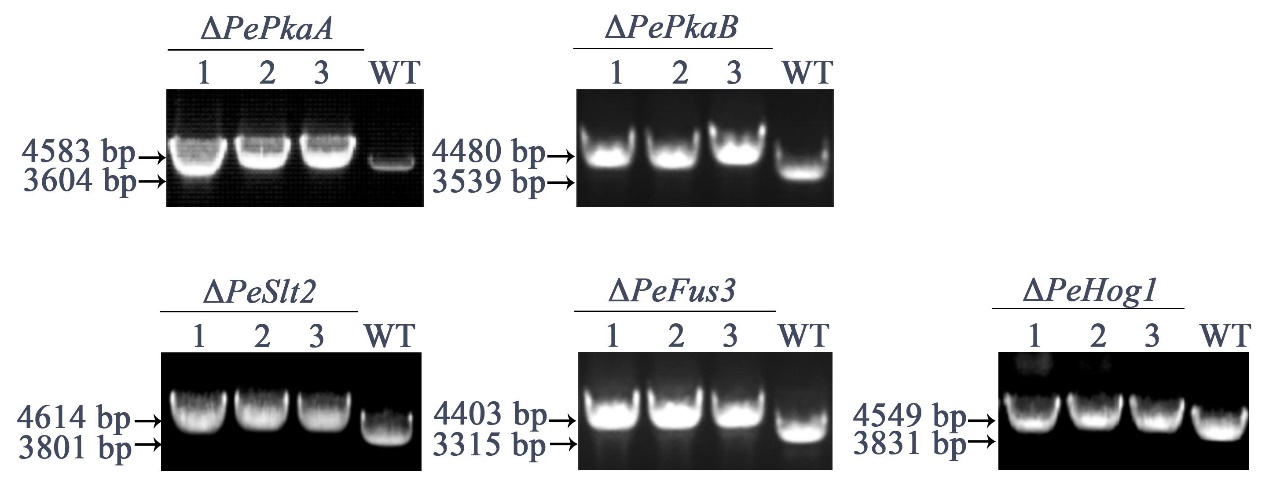
**

**Fig. S5. PCR identification of positive transformants in gene deletion strains of *PePkaA*, *PePkaB*, *PeSlt2*, *PeFus3*, and *PeHog1* in *P. expansum*.**
