# Supplementary material for: Dissecting G-protein signaling pathways in the fruit pathogen Penicillium expansum: implications for pathogenesis and patulin production
Source: Mol Hortic. 2026 Apr 8;6:28. doi: 10.1186/s43897-025-00211-w (PMC13059215; doi:10.1186/s43897-025-00211-w)
Supplement: Supplementary file 2 — Supplementary Material 2: Supplemental tables. Table S1. Primer sequences used for manipulation of G protein genes in P. expansum. Table S2. List of DEGs involved in polarized growth pathways in ΔPeGαI, ΔPeGβ, and ΔPeGγ strains. Table S3. Primer sequences used for RT-qPCR analysis of DEGs involved in polarized growth pathways. Table S4. Primer sequences used for RT-qPCR analysis of patulin cluster genes. Table S5. Primer sequences used for manipulation of PeSlt2, PeFus3, PeHog1, PePkaA and PePkaB in P. expansum. [file 43897_2025_211_MOESM2_ESM.docx]

| **Purpose** | **Primer sequences (5’ - 3’)** |
| --- | --- |
| **RT-qPCR analysis** | |
| qPeGαI-F/R | GCGCGAATACCAGCTGAAC / TGCGCTCAATGGAATCGAA |
| qPeGαII-F/R | TGAGGCCATGATGTTATTCGAA / TGATTGGTTTGCGCTTGAAC |
| qPeGαIII-F/R | AAAGCGAAGCCAACAGATTGA / ATTCTCTTCGTAGCCGCCTAGA |
| qPeGβ-F/R | TCTCAGTATCCGGCCGGTTA / TCCCATACCTTGCACTCGAAA |
| qPeGγ-F/R | ATGGCTCCCTACGAACTTCGA / CGGTCAACCGACGATATTTCA |
| **Cloning Flank L** | |
| PeGαIup-F/R | TCAGATTCCTTGAAGAACCAG / TTTGACGGTATTAAGTCGCAAT |
| PeGαIIup-F/R | CTGGGAACAAAGTGAAGATTTAGAC / GTTTTACGTCCGGTAACGACA |
| PeGαIIIup-F/R | GTTGTACGGAGTATGTAGATGTCAG / TGTGGAATCTTCAGTGGCC |
| PeGβup-F/R | AAGGGAGTCGATTTAGCTCG / TTTATCGAAACGGTACCCC |
| PeGγup-F/R | GTGGAGGACAGTCAAGGCG / CGTGACGAGTTGAGCTTTGA |
| **Cloning Flank R** | |
| PeGαIdn-F/R | TGAACCTATCGTTTGGTGATGAAG / CTCTCCGAAACAGAAAACCAACT |
| PeGαIIdn-F/R | GGTAGAGGCCCTCAATTCC / TTTGGAGGATGAGGAGGAATAC |
| PeGαIIIdn-F/R | CCGCTGAAATATACCTGAGGAC / TTGCTATGATGTGGGTGGCT |
| PeGβdn-F/R | ACCAACAAAAACAAAACGCA / ATACTCCTAGCCACTACTGACTGT |
| PeGγdn-F/R | TGCACTCACGACGGGAACT / GAGGGGGTTACTGTGGTGTG |
| **Complementation** | |
| PeGαIfl-F/R | TGAGGCGTAGACTAGTCTGATTGG / GAGCACCCACAGAATGGAACA |
| PeGαIIfl-F/R | CTGGGAACAAAGTGAAGATTTAGAC / TTTGGAGGATGAGGAGGAATAC |
| PeGαIIIfl-F/R | CGAACACCTGAGACGCAACA / CTTCATTCCTTCATTCCTGGG |
| PeGβfl-F/R | AAGGGAGTCGATTTAGCTCG / ATACTCCTAGCCACTACTGACTGT |
| PeGγfl-F/R | GTGGAGGACAGTCAAGGCG / GAGGGGGTTACTGTGGTGTG |
| **PCR detecting** | |
| pPeGαI-F/R | TCAGATTCCTTGAAGAACCAG / CTCTCCGAAACAGAAAACCAACT |
| pPeGαII-F/R | CTGGGAACAAAGTGAAGATTTAGAC / TTTGGAGGATGAGGAGGAATAC |
| pPeGαIII-F/R | GTTGTACGGAGTATGTAGATGTCAG / TTGCTATGATGTGGGTGGCT |
| pPeGβ-F/R | AAGGGAGTCGATTTAGCTCG / ATACTCCTAGCCACTACTGACTGT |
| pPeGγ-F/R | GTGGAGGACAGTCAAGGCG / GAGGGGGTTACTGTGGTGTG |
| **Southern blot probe** | |
| *hph*-F/R | TCACCCCCATCTCAACTCCA / TGCTCCATACAAGCCAACCAC |
| **Subcellular localization** | |
| gPeGαI-F/R | GGTTGCGGAATGAGCACC / TTAGATCAAACCGCAGAGACG |
| gPeGαII-F/R | GGCTGCTTAGTTTCGAAGC / TTATAGTATTAAGGTATGCAGATTC |
| gPeGαIII-F/R | GGGTCTTGTTTCAGCTCGGA / CTATAGAATACCCGAGTCTTTCAAA |
| gPeGβ-F/R | GCCGACATGAGCGGCGAA / TTACCAGGCCCAGACCTTGAG |
| gPeGγ-F/R | GCTCCCTACGAACTTCGATCC / TTACATGACGGTGCAGCAAC |

**Table S1. Primer sequences used for manipulation of G protein genes in *P. expansum*.**

**Table S2. List of DEGs involved in polarized growth pathways in Δ*PeGαI*, Δ*PeGβ*, and Δ*PeGγ* strains.**

| **Gene ID^a^** | **Annotation** | **Log2 (Fold change)** | | |
| --- | --- | --- | --- | --- |
|  |  | **WT**  **vs**  **Δ*PeGαI*** | **WT**  **vs**  **Δ*PeGβ*** | **WT**  **vs**  **Δ*PeGγ*** |
| PEG05799 | Rho guanyl nucleotide exchange factor | 0.63 | NA | NA |
| PEG10536 | Rho GTPase activator | 0.6 | NA | NA |
| PEG05752 | Rho GTPase activation protein | 1.38 | 1.39 | 1.27 |
| PEG05541 | Rho GDP-dissociation inhibitor | -0.83 | NA | NA |
| PEG10138 | GTPase Rho2 | 0.92 | NA | NA |
| PEG04751 | Ras guanine-nucleotide exchange protein | 1.82 | 1.81 | 1.51 |
| PEG10666 | ARF GTPase activator Csx2 | 0.59 | NA | NA |
| PEG01313 | GTPase-activating protein GYP7 | 0.73 | NA | NA |
| PEG08227 | GTPase activating protein Sar1 | 0.83 | NA | NA |
| PEG06073 | GTP-binding protein ypt4 | 0.59 | 0.75 | 0.83 |
| PEG01963 | Dynamin GTPase | 1.72 | NA | NA |
| PEG04978 | CDC25-like phosphatase YCH1 | -0.71 | -0.78 | -0.66 |
| PEG10119 | Guanine-nucleotide dissociation stimulator Cdc25 | -0.59 | NA | NA |
| PEG10097 | Cell division control protein 4 | 2.74 | NA | NA |
| PEG01260 | Cell cycle control protein Cdc123 | -0.74 | NA | NA |
| PEG05200 | ATP-dependent Clp protease | 0.69 | 0.85 | 0.72 |
| PEG00675 | AAA domain (Cdc48 subfamily) | NA | 1.22 | 1 |
| PEG09601 | AAA domain (Cdc48 subfamily) | NA | 1.62 | 1.26 |
| PEG01627 | Actin-related protein 2/3 complex subunit 1A | -1.95 | -1.95 | -1.83 |
| PEG00087 | Actin-related protein 2/3 complex subunit 1A | 1.16 | 1.62 | 1.46 |
| PEG00337 | SUR7/PalI family | 2.55 | 1.37 | 1.4 |
| PEG08291 | SUR7/PalI family | 1.89 | NA | NA |
| PEG08095 | HPP family protein | 5.56 | 3.16 | 3.32 |
| PEG01076 | HPP family | 1.2 | 0.62 | 0.79 |
| PEG06927 | Actin patches distal protein 1 | 2.57 | NA | NA |
| PEG09892 | Spc97/Spc98 | 0.62 | NA | NA |
| PEG05630 | Tubulin-specific chaperone D | 1 | NA | NA |
| PEG09619 | Tubulin domain | -0.94 | NA | NA |
| PEG08085 | Profilin | -0.74 | NA | NA |
| PEG10104 | Septin | 0.91 | NA | NA |
| PEG03441 | Septin | 1.37 | 1.22 | 1.03 |
| PEG07421 | actin cytoskeleton-regulatory complex protein SLA1 | NA | 1.08 | 1.18 |
| PEG07687 | Kinesin-like protein 2 | NA | -0.81 | -0.99 |
| PEG08202 | Tip elongation aberrant protein Tea4 | 0.66 | NA | NA |
| PEG11288 | Serine/threonine-protein kinase bud32 | -1.2 | NA | NA |
| PEG03281 | Cellular morphogenesis protein Bud22 | -0.84 | -0.81 | -0.85 |
| PEG06623 | Exocyst complex component 3 | -1.03 | -0.72 | -0.77 |
| PEG07607 | Chitin synthase | 2.44 | 1.67 | 1.67 |
| PEG07122 | Chitin synthase | 4.9 | 3.14 | 3.14 |
| PEG08132 | Plasma membrane stress response protein Ist2 | 1.74 | 0.67 | 0.65 |
| PEG06828 | Sodium/calcium exchanger protein | 0.6 | NA | NA |
| PEG09230 | Sodium/calcium exchanger protein | 0.75 | NA | NA |
| PEG06642 | Calcium ion transporter Vcx1 | 0.71 | NA | NA |
| PEG07104 | Vacuolar H^+^\/Ca^2+^ exchanger | -1.98 | NA | NA |
| PEG00237 | Calcineurin-like phosphoesterase | NA | 1.29 | 1.23 |

^a^Accession number from the annotated genome sequence of *P.expansum* T01 (accession no. GCA_001008385.1)

**Table S3. Primer sequences used for RT-qPCR analysis of DEGs involved in polarized growth pathways.**

| **Gene ID** | **Annotation** | **Primer sequences (5’ - 3’)** |
| --- | --- | --- |
| PEG05752 | Rho GTPase activation protein (*RhoGAP*) | ACCTCCTAAGCTGCGACGAA / TCGATCTTCCTTGCAACGAA |
| PEG04751 | Ras guanine-nucleotide exchange protein (*RasGEF*) | AACAGATGGGACAGGGCTTTT / CAGCTTTCGCTCCCGTTCTA |
| PEG00337 | *SUR7*/*PalI family* | TCTGTTCCAAATGCCGTCTTG / TTCCATAGGCCGACCTGGTA |
| PEG08132 | Plasma membrane stress response protein (*Ist2*) | CACTGGTACCGCTCGGTTTT / TTGGCACTCTAGCGTGAGCTT |
| PEG10119 | Guanine-nucleotide dissociation stimulator (*Cdc25*) | GCCCAGCTGAGAACTTTGCT / GCGGGTGTGAGCGATTAAAA |
| PEG03281 | Cellular morphogenesis protein (*Bud22*) | TCACTCTGGGCAGCATTCC / CCTCCCAAGAGGGATGCAAT |
| PEG01627 | Actin-related protein 2/3 complex subunit 1A (*Arp2/3 complex*) | CTCGCGGTCGACAAAACAA / AGGTTCGCGACCCAAGTCTT |
| PEG07687 | Kinesin-like protein 2 (*Kinesin-2*) | GGTCTACATCCGTGGCGAAA / TCCAACATGCCTTCGTAGCA |

**Table S4. Primer sequences used for RT-qPCR analysis of patulin cluster genes.**

| **Gene** | **Primer sequences (5’ - 3’)** |
| --- | --- |
| *PatA* | AAAGGCCGGTGCATTGATC / TTGGAGGCTTTGGTGAGCAT |
| *PatB* | GCCAGGCTATGCGATTGAGT / GCTGGAACCCTGTCCATTGT |
| *PatC* | TCCACCTGCGAATATCCCTTA / CATCGCCAGTGCCATTTTC |
| *PatD* | ATGAGATTCGTCTGCGCAAAG / CTACCCAAGCGGGATGAGATT |
| *PatE* | CATTCTCATCGGGCCTGAGT / TCGAAGCTCTTCCGGACATG |
| *PatF* | GCGAGTGAATTCGGCCAAT / GTCCGACCCAAAGGATGAAG |
| *PatG* | CGGCCGTCTTGAAGGAAAT / CTTGCCGTAGCGGGTGAATA |
| *PatH* | CATTTATCGGCGGTGTTCTGA / GATCAACGCTTGCACGATAGC |
| *PatI* | GCAAACTCATTCCGCAAGGA / TGGTTCTTGCCATCGATCAC |
| *PatJ* | CGCCAGACATACCGCCATA / TTTGGTCGATCGGGACTGTT |
| *PatK* | GACGCTGGGCTACTGGATTG / TCGTGCGTGAGGCCAGTAT |
| *PatL* | GCAGGAGATCCGTTTCAGACA / CCACTGACCGACGGTTACAAC |
| *PatM* | ACCCACAGCTGCACATGGA / AGCGAGAAGAGGCGGAAGA |
| *PatN* | CGTTCGATGTCGCTAGCAAA / GGCGATAATCACGTCAATTCG |
| *PatO* | TCGCCTCCTGGTGTGTATCTT / AAGCGTGCCCAGTCATTCAG |
| *β-tubulin* | CTCCAGCTCGAGCGTATGAAC / GGCTCCAAATCGACGAGAAC |

**Table S5. Primer sequences used for manipulation of *PeSlt2, PeFus3, PeHog1, PePkaA and PePkaB* in *P. expansum*.**

| **Primers** | **Primer sequences (5’ - 3’)** |
| --- | --- |
| **Cloning Flank L** | |
| PeSlt2up-F/R | GGAACCAACTGTTATGGAGGG / GGCGAGTTGTCGCGTCGTA |
| PeFus3up-F/R | CCATCAAACCAGAGAAAGCAGA / GTCGATAACGGTTTCTTTGGTAG |
| PeHog1up-F/R | TCACCCTCTCAACCCGTCA / CATCGGAGATAATATGGAAGTAA |
| PePkaAup-F/R | GCCACTGGTGCTTTCTTTTC / GGAGAGAAGTTTAGACAGGCTTT |
| PePkaBup-F/R | ATGGACAGGACGGTAGCTAACT / TAGAATGGTCAAAGATCATGTCG |
| **Cloning Flank R** | |
| PeSlt2dn-F/R | TACCTTTTTATCTTTTTCTGCCTG / CCATCTGTGACCTGTAAGAGGG |
| PeFus3dn-F/R | TCTTTTCTTGCAATTTACTAACCTT / CATATACAACCCATCCCACACC |
| PeHog1dn-F/R | GCGGCAATCCACATACCTCT / CTCAAAAACGCCTATTTCCACA |
| PePkaAdn-F/R | GGTGCTTGTATGATTTTATGACG / GAATAGGCGAAGTTCCTGAG |
| PePkaBdn-F/R | AAGATTTCTTTGCCATTACAGC / GCTTCTATATGTTTCTACCGAACT |
| **PCR detecting** | |
| pPeSlt2-F/R | GGAACCAACTGTTATGGAGGG / CCATCTGTGACCTGTAAGAGGG |
| pPeFus3-F/R | CCATCAAACCAGAGAAAGCAGA / CATATACAACCCATCCCACACC |
| pPeHog1-F/R | TCACCCTCTCAACCCGTCA / CTCAAAAACGCCTATTTCCACA |
| pPePkaA-F/R | GCCACTGGTGCTTTCTTTTC / GAATAGGCGAAGTTCCTGAG |
| pPePkaB-F/R | ATGGACAGGACGGTAGCTAACT / GCTTCTATATGTTTCTACCGAACT |
